# Pollen metabarcoding reveals a broad diversity of plant sources available to farmland flower visitors near tropical montane forest

**DOI:** 10.3389/fpls.2024.1472066

**Published:** 2025-01-07

**Authors:** B. Karina Montero, Nicole Gamboa-Barrantes, Geovanna Rojas-Malavasi, E. Jacob Cristóbal-Perez, Gilbert Barrantes, Alfredo Cascante-Marín, Paul Hanson, Manuel A. Zumbado, Ruth Madrigal-Brenes, Silvana Martén-Rodríguez, Mauricio Quesada, Eric J. Fuchs

**Affiliations:** ^1^ Centro de Investigación en Biodiversidad y Ecología Tropical, Universidad de Costa Rica, San José, Costa Rica; ^2^ Escuela de Biología, Universidad de Costa Rica, San José, Costa Rica; ^3^ Laboratorio Nacional de Análisis y Síntesis Ecológica, Escuela Nacional de Estudios Superiores, Unidad Morelia, Universidad Nacional Autónoma de México, Morelia, Michoacán, Mexico; ^4^ Laboratorio Binacional de Análisis y Síntesis Ecológica, Universidad Nacional Autónoma de México - Universidad de Costa Rica (UNAM-UCR), Morelia, Michoacán, Mexico; ^5^ Investigador Colaborador, Museo de Zoología, Universidad de Costa Rica, San José, Costa Rica; ^6^ Laboratorio de Ecología Evolutiva de Plantas, Escuela Nacional de Estudios Superiores-Morelia, Universidad Nacional Autónoma de México, Morelia, Michoacán, Mexico

**Keywords:** insect visitor communities, low-intensive farming, fruit crops, tropical montane forest, ecosystem services

## Abstract

Despite the widely recognized role of pollinators in ecosystem services, we currently have a poor understanding of the contribution of Natural Protected Areas neighboring agricultural landscapes to crop pollinator diversity and plant-pollinator interactions. Here, we conducted monthly surveys over a period of one year to study the diversity of insect visitors in dominant fruit crops—avocado, plum, apple, and blackberry—and used pollen DNA metabarcoding to characterize the community of plant sources in and around low-intensive farmland bordered by protected montane forest in Costa Rica. We found that crops and native plants had distinct communities of flower visitors, suggesting the presence of fine-scale habitat differences. DNA metabarcoding coupled with a custom-built reference database, enabled us to identify plant sources among pollen samples with high taxonomic resolution (species or genus level). We found that insect visitors carried pollen from a large diversity of plant taxa, including species native to the montane forests and highland páramos of Costa Rica. The diversity and composition of plant sources were variable across fruit crops and insect groups. Wildflower visitors such as bumblebees and syrphid flies, use a diverse range of plant taxa at similar levels to managed honeybees. This indicates the potential contribution of a diverse community of insect visitors to the pollination services of fruit crops and native flora. Overall, our study suggests that low-intensive farming practices that promote the presence of common ruderals combined with nearby protected forests contribute to maintaining diverse insect communities that provide crucial pollination services.

## Introduction

The expansion of farmland and urban areas has led to an accelerated biodiversity loss, threatening the provisioning of ecosystem services (Vanbergen and Insect Pollinators Initiative, 2013). Safeguarding pollination services has become a priority issue since a large and increasing number of crops depend on biotic pollination (Aizen et al., 2019; Ashworth et al., 2009; Klein et al., 2006; Novais et al., 2016). Tropical regions proportionally account for a larger production area of pollinator-dependent crops compared to temperate regions (Aizen et al., 2008; Porto et al., 2021); however, the potential role of wild pollinator communities in providing ecosystem services to tropical agricultural crops remains poorly understood.

Natural habitats act as reservoirs of diverse communities of pollinators, which may provide an important ecosystem service to neighboring farmlands by increasing crop yield (Carvalheiro et al., 2011). Pollinator diversity can increase the time span and number of flowers visited (Fründ et al., 2013; Hoehn et al., 2008) contributing to the stability of plant-pollinator interactions (Garibaldi et al., 2011). Wild bees, for instance, are the main pollinators of economically important crops such as coffee (Klein et al., 2003; Ricketts et al., 2004), watermelon (Kremen et al., 2002; Winfree et al., 2007), and mango (Carvalheiro et al., 2012). For other crops, like avocado, hoverflies are the most abundant and effective pollinators (Celis-Diez et al., 2023). Furthermore, whilst managed honeybees play a crucial role in crop production (Morse and Calderone, 2000), their wild counterparts have been shown to be efficient pollinators (Freitas and Paxton, 1998; MacInnis and Forrest, 2019; Pérez-Méndez et al., 2020). Our study aims to improve our understanding of the ecosystem services provided by wild insect flower visitors in highland tropical farmland. We studied the community of insects visiting four dominant fruit crops—avocado, plum, apple, and blackberry—growing in the valley of San Gerardo de Dota in the highlands of Costa Rica. Local fruit crop farmers conduct infrequent mowing as an alternative to applying herbicides, plant a mix of native and introduced species alongside crops to prevent erosion and promote pollination, and have recently (during the last 10 years) reduced the use of pesticides as a measure to prevent the loss of biodiversity (personal communication from local farmers). Moreover, this region is characterized by low-intensity fruit farming surrounded by a protected area of montane forest, Los Quetzales National Park.

As a first objective, we characterized the diversity and composition of insect communities visiting fruit crops and wild herbaceous and shrubby plants in San Gerardo. The presence of native flowering plants close to and within crops can increase pollinator pools, provide semi-natural habitats that enhance pollinator movement (Krimmer et al., 2019), increase the likelihood of pollen-mediated gene flow (Cane and Love, 2018), and positively impact crop productivity (Carvalheiro et al., 2012). Evaluating their contribution to maintaining insect communities will enable us to identify management and conservation strategies (Carvalheiro et al., 2011) to promote pollination services in local fruit crops.

Our second objective was to determine the diversity of floral resources used by the insect communities visiting fruit crops. Plant-pollinator interactions are crucial to the stability of pollination systems (Huang et al., 2021). However, flower visitation surveys provide a partial understanding of plant-pollinator interactions, focused mainly on the perspective of one end of the interaction, i.e., the plant, rather than both interaction partners (Bosch et al., 2009). To gain a better understanding of the pollinator perspective, we complement visitation surveys with pollen identification using DNA metabarcoding (Bell et al., 2017; Richardson et al., 2015). This approach enabled us to evaluate the diversity of plants visited by the insect community in fruit crops, thus providing some insight into the contribution of wild native plants to maintaining pollination services.

## Materials and methods

### Study area

We conducted fieldwork in the valley of San Gerardo de Dota (9°33´´N, 83°47´W, 2300 masl), located in the Pacific slope of the Talamanca Mountain Range, San José province, Costa Rica. The upper montane cloud forest of the Talamanca Mountain range in Costa Rica is an evergreen forest dominated by oaks (*Quercus* spp.). Epiphytes (including bromeliads, orchids, mosses and lichens), tree ferns, and bamboo form a characteristic component of the montane forest vegetation (Kappelle et al., 1992). Daytime average temperature at the study site is 17°C with a median annual precipitation of 2,500 mm. This region experiences two seasons: a dry season from December to March and a wet, rainy season from April to November.

**Figure 1:**
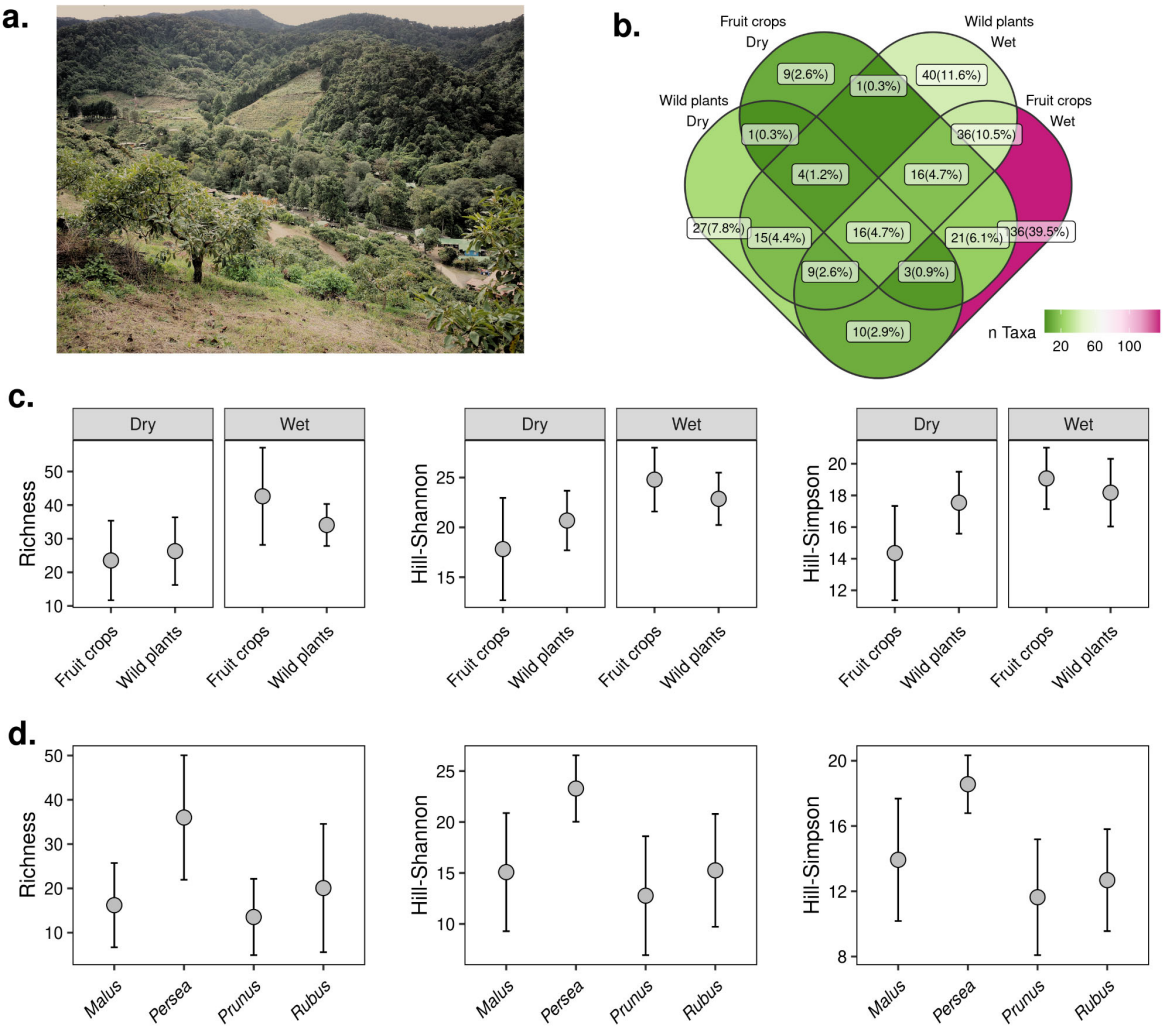
Diversity of insect visitors in fruit crops and wild plant transects in San Gerardo de Dota. **(A)** The valley of San Gerardo is surrounded by protected areas (in the picture, the forest in the background corresponds to Los Quetzales National Park) with fruit crops growing on the hills surrounding the valley. Avocado plantations (foreground) are the dominant fruit crop in the study site. **(B)** Venn diagram showing the number (and percentage) of taxa (lowest taxonomic assignment) shared among visitors in fruit crops and wild plants during the dry and rainy seasons. **(C)** Hill-diversity estimates of insect visitors between fruit crops and wild plants during the dry and wet seasons. **(D)** Hill-diversity estimates among the four fruit crops (apple (*Malus domestica*), avocado (*Persea americana*), plum (*Prunus domestica*), and blackberry (*Rubus* spp.)) studied in San Gerardo. Error bars correspond to 95% CI.

The valley of San Gerardo is flanked by Los Quetzales National Park, Tapantí National Park, and Los Santos Forest Reserve. Most human activities in San Gerardo occur in the upper basin of the Savegre River (**Figure 1A** illustrates the presence of montane forest on the hilltops of San Gerardo and fruit crops in the valley). Low-intensity fruit orchards (apple and peach) were planted to replace pastures in the 1980’s. Avocado plantations were established at the beginning of the 1990’s. Currently, avocado is the dominant fruit crop, followed by apple, blackberry, and remnant patches of plum trees. Ecotourism is the predominant economic activity in San Gerardo de Dota, which encourages farmers to minimize the use of agrochemicals and to attract avifauna and insects by means of ornamental gardens and native trees (e.g., *Ocotea* sp.). Farmers perform mowing every two months to manage weed growth.

### Flower visitation surveys

We conducted monthly surveys to estimate the diversity of insect visitors to the flowers of local native plants, focusing on herbs and shrubs, including ruderals commonly found growing within the fruit crops. The four transects were about 150 m in length and 4km away from the fruit farms, respectively. We sampled insect visitors of avocado (2 farms), apple (2 farms), plum (1 farm), and blackberry (2 farms) (**Supplementary Table 1**). Two farms (Lauraceas and Sueños del Bosque) rent *Apis mellifera* apiaries from October to February. The farms were roughly 4 km apart from each other. We visited farms during the peak blooming periods of each fruit crop. Crop and wild plant surveys consisted in collecting all insects in a focal flowering plant or tree during a 15 min period. We collected insects on flowers using handheld nets or by positioning a clean transparent plastic bag on the plant and directing the insects into the bag. Insects were then stored in collection tubes with 70% ethanol for later identification in the lab. We carried out surveys during the morning hours of clear, warm days, avoiding rainy and windy days. Surveys lasted between 2 and 8 hours, depending on weather conditions and flower availability.

Insects were identified by trained taxonomists and specialists (G. R-M., N. G-B, P. H., and M. A. Z.) using available keys (Brown et al., 2009; Brown et al., 2010; Michener et al., 1994) and, when needed, comparing specimens collected with those deposited in entomology collections, namely the Museo de Zoología, Universidad de Costa Rica (UCR), and the Museo Nacional de Costa Rica. Most tropical insects lack taxonomic keys at the species level, and a considerable number of these insect species are undescribed; thus, many of the species collected in this study were assigned to morphospecies based on the criteria of expert taxonomists. We deposited voucher specimens in the Museo de Zoología (UCR). A subset of 242 insects collected from the fruit crops were further processed to isolate DNA from the pollen grains attached to the insects. Collection tubes were stored at -20°C in the lab at UCR until further processing.

### Pollen DNA metabarcoding

#### Sample processing

We used 242 pollen samples to extract DNA for metabarcode analysis. Due to the low amount of pollen carried by Diptera species, we combined pollen from at least three individuals of the same species or morphospecies for a given sampling session to obtain adequate pollen quantity. The number of pooled individuals from each species or morphospecies varied according to the species and size of the insect species, as well as the amount of pollen observed on its body. For example, for small-sized Syrphidae and Muscidae, we pooled samples from 3–5 individuals of the same species. Pollen grains were removed from the insect’s body using alcohol and a cotton swab following previously published protocols (Klečka et al., 2022; Suchan et al., 2019). Then the cotton swab was soaked in a 1.5 ml Eppendorf tube filled with 0.5 ml of 95% alcohol and pressed against the inner wall to release pollen grains. The pollen mixture was centrifuged at 14 000 rpm for 10 minutes, leaving a visible pellet at the bottom of the tube. We removed the supernatant ethanol by pipetting the liquid without disturbing the pollen pellet and allowed the residual ethanol to evaporate at room temperature for 24 hours under a chemical hood. Samples were incubated at 60° C and shaken at 900 rpm for 1h with 500 *μ*l of Cell Lysis Solution and 10 µl of Proteinase K (Qiagen, Hilden, Germany). Pollen grains were ground using a TissueLyser II (Qiagen, Hilden, Germany) with a mixture of silica beads of the following sizes: 106µ (30%), 150-212µ (50%), 212-300µ (10%), and 425-600µ (10%), using four cycles of 1 min at 30 Hz. DNA was extracted using the protocol of the Wizard^®^ Genomic DNA Purification Kit (Promega) for plant tissue, with the following modifications: a) the time of incubation with Nuclei Lysis Solution was extended to 1 hour, and b) the precipitation with isopropanol was extended overnight at -20°C. DNA extractions were quantified with a Quantus Fluorometer (Promega).

#### Amplicon sequencing

We characterized pollen diversity by amplifying a fragment of the ITS2 region (Cheng et al., 2016) using the primers ITS2p4_f: “YGACTCTCGGCAACGGATA” and ITS2p4_r: “CCGCTTAKTGATATGCTTAAA”. The ITS2 region was amplified using a PCR reaction with a total volume of 30 µl, which included 15 µl of PlatinumTM SuperFiTM PCR Master Mix (Invitrogen), 0.5 M of the forward and reverse primers, and approximately 10 ng of DNA template. We used a Veriti 96-Well Thermal Cycler (Applied Biosystems) with the following cycle conditions: 3 minutes at 98°C denaturation, 30 cycles of 30s at 98°C, 30s at 60°C, and 30s at 72°C, followed by a five-minute final extension step at 72°C. Samples with a clear band on a 2% agarose gel were purified using magnetic beads following the Pronex (Promega) and concentrations were quantified with a Quantus fluorometer. Subsequently, we used Nextera XT indices (Illumina) to tag with identical dual indexes for all amplicons. This was done in accordance with the Metagenomic Library Sequencing Preparation Protocol from Illumina, which was executed using the Qiagen Multiplex PCR Kit. The indexed libraries were normalized to 4 nM, pooled, and sequenced on MiSeq Illumina (2 x 250 pair-end) at the Unidad de Secuenciación Masiva of the Instituto Nacional de Medicina Genómica (INMEGEN), México.

#### ITS2 data processing

The data generated by amplicon sequencing of the ITS2 marker was processed with the DADA2 algorithm (Callahan et al., 2016) implemented in the R program, version 4.2.2 (R Core Team, 2023) using the libraries *dada2* (Callahan et al., 2016), *ShortRead* (Morgan et al., 2009), *Biostrings* (Pagès et al., 2023) and *stringr* (Wickham and RStudio, 2022). Primers from the demultiplexed reads were trimmed with the external tool *cutadapt* (Martin, 2011) using the sequences of the primers as cut points. Low-quality reads were filtered based on the parameters minLen = 50, maxLen = 600, maxN = 0, maxEE = c(2,2), and truncQ = 2. The learnErrors function was then used to generate a parametric model of the error in the data. DADA2 estimates the error rate of sequenced samples to probabilistically discriminate sequences that are the product of real variant sequencing errors. Using this model, inference of the assigned sequence variants (ASVs) was then performed using the dada function (Callahan et al., 2016).

We constructed an ITS2 reference database following the DB4Q2 pipeline (Dubois et al., 2022) to assign taxonomy to ASVs. The DB4Q2 pipeline generated a curated baseline, which was imported into the qiime2 environment (Bolyen et al., 2019). To ameliorate the limitations of taxonomic classification in metabarcoding studies (Arstingstall et al., 2021), we created a local database of ITS2 sequences consisting of plant specimens collected at our sampling site as well as other common and endemic species of the montane forest and paramo ecosystems. We sequenced a total of 140 species from 102 genera across 57 families of plants (see **Supplementary Text S1** for details). We manually merged both ITS2 sequence databases (i.e., sequences from NCBI and the local database) to create the RDP trained classifier file to assign taxonomy using a native Bayesian method (Wang et al., 2007) implemented in the dada2 library using the assign Taxonomy function. Taxonomic predictions have been previously shown to be influenced by user-defined choices when processing and curating sequence data of custom-built databases (Dubois et al., 2022). Critically, the impact of these choices is often project-specific. For this reason, we built a total of 16 databases and explored their performance with regard to the fraction of reads assigned to plant taxonomy. A detailed description of database processing parameters is provided in **Supplementary Text S1**.

Sequence variants were classified and binned to the lowest taxonomic level (i.e., genus or species). We removed ASVs with poor taxonomic resolution (i.e., ASVs classified to family or above) for downstream analysis. The dataset was then transformed into presence/absence data.

### Statistical analyses

#### Diversity and composition of insect visitors

We characterized diversity based on incidence data across surveys, which were considered units of replication (i.e., relative number of species detected across fruit crops and wild plant transects). We summarized the occurrences of each taxon per sampling unit and used the presence-absence matrix as input in the function iNEXT to calculate sample completeness and in the function estimateD to compute coverage-based Hill-diversity estimates using the iNEXT package (Chao et al., 2014; Hsieh et al., 2016).

Coverage-based estimators were used to adjust for differences in sampling time among sites and seasons. We used three measures of Hill diversity (richness, Hill-Shannon, and Hill-Simpson) to provide insight into evenness and dominance patterns in insect visitor communities (Alberdi and Gilbert, 2019a; Roswell et al., 2021). Coverage-based analyses of Hill-diversity and associated 95% confidence intervals (CI) enabled us to compare alpha diversity of insect visitors between fruit crops and wild plants and between the dry and wet seasons. We also tested for differences in insect visitor diversity across fruit crops in San Gerardo. Non-overlapping 95% CI indicated significant differences among groups.

We used the permutational analysis of variance (PEMANOVA) (*vegan::adonis2*) to test for differences in insect visitor community composition between fruit crops and wild plants during the dry and wet seasons, and differences among fruit crops. We used analysis of variance (ANOVA) to test for equal dispersion in the data (i.e., homogeneity of multivariate variances) using the *vegan::betadisper* function. We found differences in the overall dispersion between fruit crops and wild plant data (F = 9.76, P = 0.003) and between the dry and wet seasons (F = 14.08, P = 0.004). However, dispersion was similar when comparing individual fruit crops and wild transects (F = 1.21, p = 0.32). For consistency, we used Hill-number dissimilarity measures for the analysis of community composition. Specifically, we calculated Sørensen dissimilarity (1-*C_qN_*) as a measure of overlap using the function pair_dist for *q* values 0, 1, and 2, implemented in the hilldiv package (Alberdi and Gilbert, 2019b). We used non-metric multidimensional scaling (NMDS, *vegan::metaNMDS*) to visualize the extent of differences in the composition of insect visitors between seasons and across fruit crops and wild plant transects.

#### Diversity and composition of plant sources

We explored the patterns of diversity in pollen samples based on fruit crop type and insect visitors. For the latter, we grouped samples in five categories: the managed honey bee *Apis melifera*, bumblebees *Bombus ephippiatus*, hoverflies (Syrphidae), non-syrphid flies and other insect visitors (Hymenoptera genera *Partamona* and *Lasioglossum*, and the Coleopteran *Astylus*). For analysis, we used the same statistical approach as with insect visitor diversity using Hill-numbers on plant species (or genera) incidence data.

## Results

A main objective of our study was to characterize the community of wild flower visitors in fruit crops surrounded by montane forest. Overall, we conducted 48 sampling sessions (234 hours) between June 2021 and November 2022, 28 (137 hours) in the fruit crop farms and 29 (97 hours) in the wild plant transects (**Supplementary Table 2**). We identified 46 species from 23 families of flowering herbaceous plants and shrubs (full list of species and flowering dates may be found in **Supplementary Table 1**), the majority of which belong to the Asteraceae (30%). Close to half (46%) of the plants surveyed in the wild plant transects also occur in the fruit crops (**Supplementary Table 1**).

We recorded a total of 1303 insect visitors along the wild plant transects and 2806 in the four fruit crops. The most abundant visitors were managed honeybees (17%), followed by syrphid flies (16%), sweat bees (*Lasioglossum* sp., 5%), and bumblebees (5%) (see details of insect taxonomic classification in **Supplementary Table 3** to **Supplementary Table 8** and representative taxa shown in **Supplementary Figure 1**). Most taxa were identified to species or morphospecies (n = 3313, 81%). Overall, we identified 144 genera in 94 families from 5 insect orders. We removed individuals unassigned to family level (n = 77, 1.9%) and used the highest taxonomic assignment of an individual for analyses.

### Diversity of insects visiting fruit crops and wild plant flowers

We used Hill-diversity estimates to describe the diversity patterns of flower visitors according to season and site (i.e. fruit crops and wild plant transects). A larger number of taxa (40%) were unique to fruit crops sampled during the rainy season compared to the dry season (3%) (**Figure 1B**). We found that the diversity of flower visitors was similar in fruit crops compared to wild plant transects, a result that was consistent across seasons and Hill-diversity estimates (**Figure 1C**; **Supplementary Figures 2**, **3**). Fruit crops flowered at different times of the year. Avocado trees had the most extended flowering periods, followed by blackberry, apple, and plum crops (**Supplementary Table 2**). Apple and plum varieties planted in the region bloom twice a year (in July-August and January), with short (approx. 1 month) windows of flowering bouts. We observed that avocado plantations differ from the other fruit crops in that they tend to attract a higher diversity of insect visitors during the flowering period although diversity was not significantly different across fruit crops (**Figure 1D**; **Supplementary Figures 4**, **5**).

**Figure 2:**
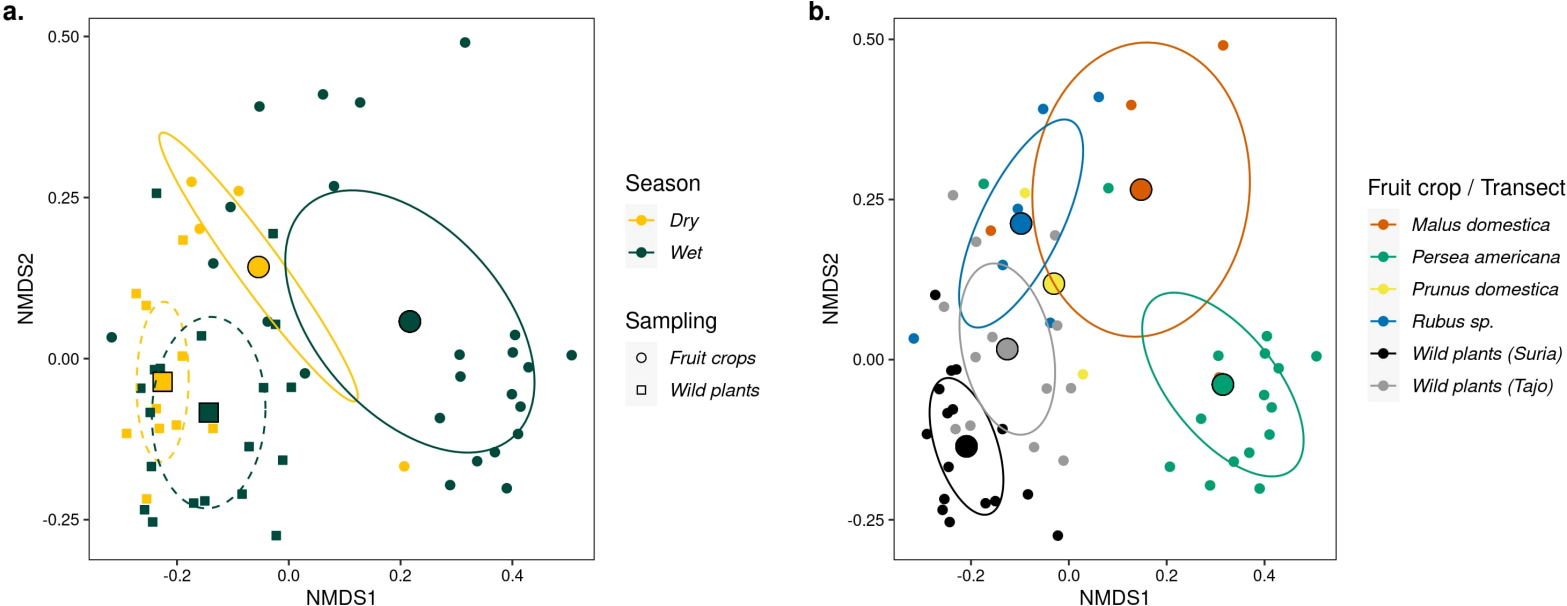
Non-metric multidimensional scaling (NMDS) of insect visitor composition using the Hill number Sørensen-type dissimilarity. **(A)** Clustering of sampling units according to whether insect visitors were sampled in fruit crops (filled dots) or in wild plant transects (filled squares), and clustering according to the dry or wet season, designated with yellow and green colors, respectively. Ellipses denote the standard error of the centroid insect community for fruit crops (solid lines) and wild plants (dotted lines) at 95% confidence. **(B)** Distinct community composition across fruit crops and wild plant transects, each designated with different colors. Ellipses denote 95% confidence intervals. Note that for plum, the sample size was insufficient to draw an ellipse (N _sampling units_ = 2).

We found that there is little overlap in the composition of insect visitors between fruit crops and wild plants (*C_q_*_=1N_: F = 7.18, p < 0.0001, *R* ^2^ = 0.11) and a significant, albeit weaker effect of season (*C_q_*_=1N_: F = 2.42, p = 0.004, *R* ^2^ = 0.04) (**Figure 2A**). Results were consistent across estimates based on the three Hill *q* levels analyzed (**Supplementary Figure 6**). Furthermore, we found a strong effect (*R* ^2^ = 0.24) of type of fruit crop on community composition (F = 2.51, p < 0.0001), suggesting fruit crop-specific insect communities (**Figure 2B**; **Supplementary Figure 7**).

### Sequencing of pollen DNA from insect visitors

We amplified and sequenced the ITS2 region of 157 out of 242 pollen samples (65%) to characterize the plant sources found in flower visitors of fruit crops. Obtaining high quality DNA from pollen samples for sequencing was a major challenge in our study; 12% of samples yielded no DNA, 7% did not amplify, and 15% were not indexed for sequencing due to very low concentrations in the first step of PCR. Close to half of the pollen samples that failed to yield high-quality DNA were from insects in the order Diptera (49%, all collected in the avocado farms), followed by *Apis* (27%), and Coleoptera (10%). The low amount of pollen grains attached to the bodies of dipterans may have been the main limiting factor in obtaining sequencing data for this group. As stated in the previous section, we found a higher diversity of insect visitors in avocado trees compared to the other fruit crops. Hence, the number of insect taxa used to study the diversity of plant sources via pollen metabarcoding from fruit crop insect visitors was greater for avocado, particularly for Diptera, since taxa in this group were diverse and abundant (**Supplementary Table 9**).

We detected 2,387 sequence variants in a total of 3,064,745 quality-filtered, denoised, and chimera filtered sequencing reads with an average sequencing depth of 18,508 reads per sample (range 880 - 40,627). In our study, a reference database that incorporated local diversity and built from sequences specific to the Neotropics (i.e., with a geographic restriction applied to sequence retrieval from NCBI, **Supplementary Text S2**) yielded the highest proportion of reads assigned to genus (95%) and to species (85%) (**Supplementary Figure 8A**). We did not observe an impact of dereplication (i.e., merging identical sequences with identical taxonomic annotations into a unique sequence, **Supplementary Text S1**) but extracting primer-specific amplicons can result in a low fraction of reads with taxonomic assignment (**Supplementary Figure 8**).

**Figure 3:**
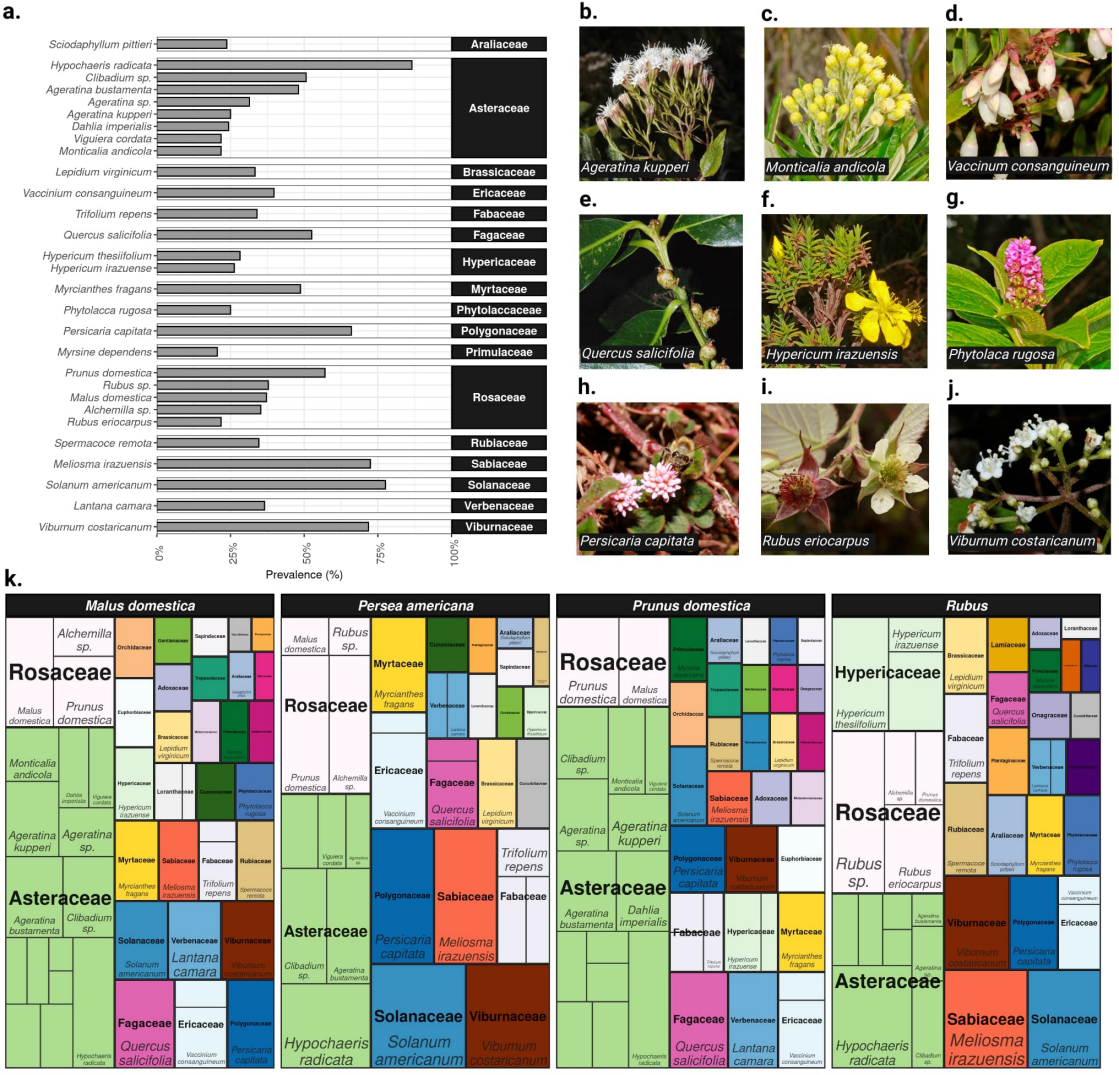
Plant sources detected via pollen metabarcoding. **(A)** Barplot showing the prevalence of common plant taxa (> 20%) across all pollen samples. **(B–J)** Examples of plant species identified in the pollen carried by flower visitors. Photo credits: Alfredo Cascante-Marín **(K)** Treemap showing the composition of plant taxa (> 3% within fruit crop prevalence) found in each fruit crop. Each tile represents a plant species (or genus), with labels shown for common species only (>20%). The area of the tile and label font size is proportional to the within fruit crop prevalence. The fill colors correspond to plant families and family labels are in bold typeface.

Among the 2,387 pollen sequence variants, we identified a total of 153 species of plants, spanning 140 genera, 68 families, and 37 orders. Of these, 29 plant taxa (25 of which were identified to species and 4 to genus level) were relatively common (i.e. > 20% prevalence) (**Figures 3A–K**). A large proportion of plant taxa were rare (i.e., close to half (48.7%) of plant taxa were found in up to two samples). Moreover, we identified a mean of 18.5 (range 3 - 48) plant taxa per sample (**Supplementary Figure 9**). We found that two endemic plant species, *Meliosma irazuensis* (Sabiaceae, 72.4%) and *Viburnum costaricanum* (Adoxaceae, 71.8%), and other herbaceous plants, such as flatweed (*Hypochaeris radicata*), glossy nightshade (*Solanum americanum*), and pink knotweed (*Persicaria capitata*), were among the most common plant species in pollen samples (> 65% prevalence) (**Figure 3A**). Other endemic plants were also commonly detected, including *Hypericum irazuense* (Hypericaceae, 26.3%) and *Diplostephium costarricense* (Asteraceae, 18.6%). The oak species *Quercus salicifolia*, native to central Mexico and Central America, featured among the most frequent plant species (52.56%) recovered from pollen samples (**Figure 3A**). Regarding pollen from the fruit crops, we found that plum (57.1%), followed by blackberry (37.8%) and apple (37.1%) were more commonly detected as plant sources, compared to an observed low prevalence of avocado (0.04%).

### Diversity of plant sources among insect visitors of fruit crops

**Figure 4:**
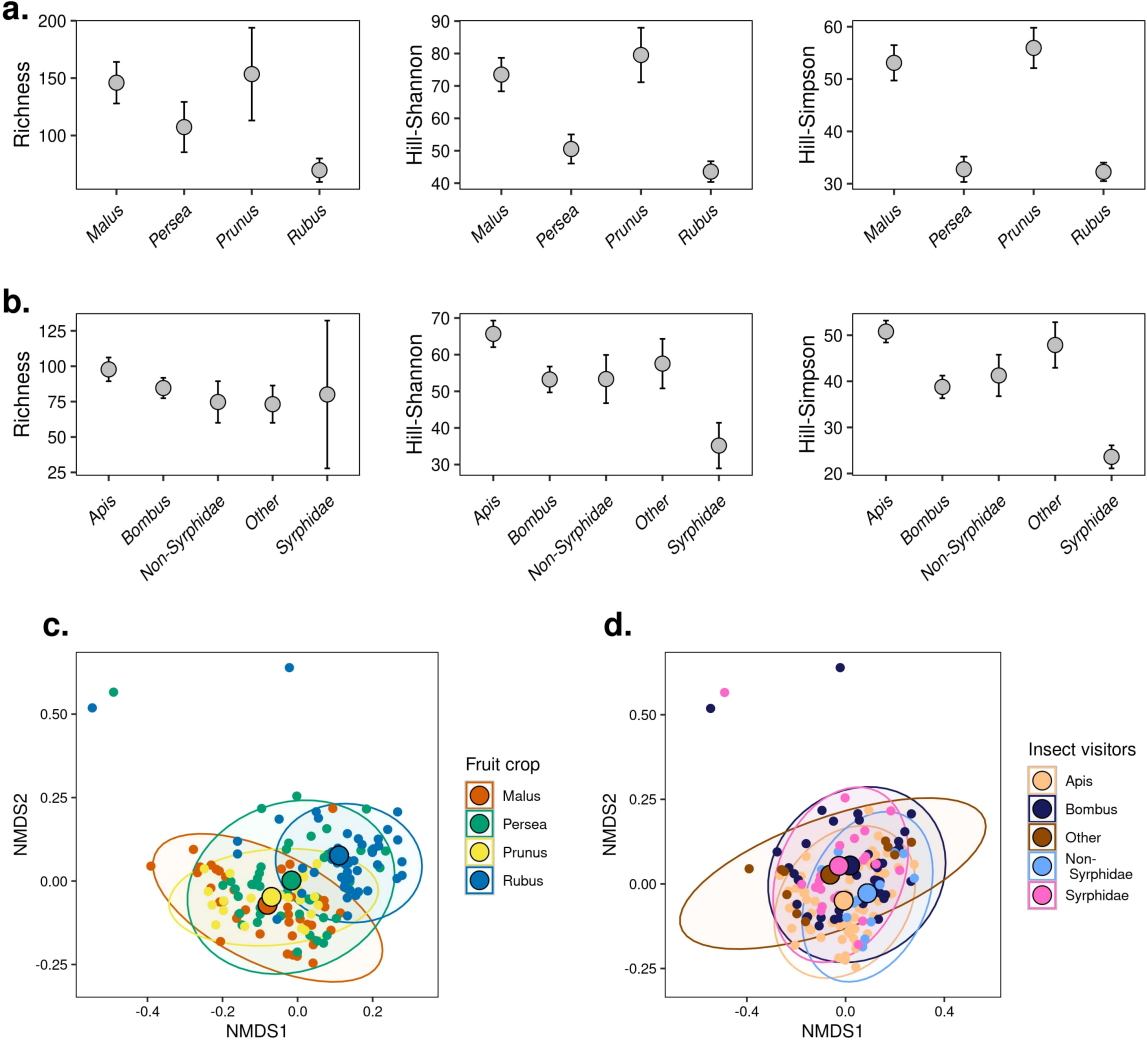
Alpha and beta diversity of plant species detected by pollen metabarcoding. Hill-number diversity estimates according to fruit crop **(A)** and insect visitor group **(B)**. Error bars correspond to 95% CI. Diversity estimates are based on coverage. NMDS (stress _q1_ = 0.126) showing the differences in composition of plant species according to fruit crop **(C)** and insect visitor group **(D)** recovered from pollen metabarcoding using the Hill number Sørensen-type dissimilarity.

We used incidence data to test for differences in alpha diversity of plant species (or genera) identified in the pollen samples. Sampling completeness was relatively consistent across the four fruit crops (range: 94% - 99%, **Supplementary Figure 10**) and the groups of insect visitors (range: 89% - 97%, **Supplementary Figure 12**) studied in San Gerardo. We found that the diversity of plant sources according to richness was similar across fruit crops and across insect visitors (**Figures 4A, B**, overlapping CI in q = 0 in **Supplementary Figure 11** and **Supplementary Figure 13**). Richness error estimates were particularly high for pollen samples collected from syrphid flies, likely due to pooling individual flies for pollen analyses (**Figure 4B**). In contrast to richness estimates, the patterns of diversity, according to Hill-Shannon and Hill-Simpson, revealed consistent differences between crops. Estimates of evenness in apple and plum crops were higher compared to avocado and blackberry (**Figure 4A**; **Supplementary Figure 11**). These results suggest that in avocado and blackberry farms, insects visited fewer dominant plant taxa compared to the other fruit crops. We found a similar diversity of plant sources across insect groups according to Hill-Shannon estimates, but Hill-Simpson estimates revealed lower diversity in syrphid flies compared to all the other insect groups (**Figure 4B**; **Supplementary Figure 13**).

With respect to patterns of plant species composition, we found that fruit crops were a stronger predictor (*C_q_*_=1N_: F = 7.75, p < 0.0001, *R* ^2^ = 0.13) compared to insect group (*C_q_*_=1N_: F = 2.39, p < 0.0001, *R* ^2^ = 0.059). We observed that pollen samples from plum and apple farms cluster closer together, while pollen samples from blackberry appear to have a distinct composition of plant species. The community of plant taxa in avocado seems to be highly heterogeneous (**Figure 4C**). We found a large overlap in plant sources among the insect groups (**Figure 4D**; **Supplementary Figure 14**).

## Discussion

Crop flower visitation by wild insects is considered a reliable proxy for pollination services (Garibaldi et al., 2013); however, patterns of wild pollinator diversity in tropical agricultural landscapes are relatively scarce. We found a species-rich community of insect visitors that carried pollen from a large diversity of plant taxa, including species native to the montane and páramo ecosystems of Costa Rica’s highlands. Together, these results suggest that current farming practices as well as proximity to protected areas likely contribute to maintaining pollination services in the region.

### Diversity of fruit crop and wildflower visitors

We found that fruit crops attract a large diversity of insect pollinators. These farming practices appear to have a positive impact on pollinator diversity by promoting the presence of native plants that provide additional food sources that attract a diverse community of pollinators. Indeed, we observed that close to half of the species of herbaceous and ruderal plants surveyed in the wild plant transects were also present in fruit crop farms.

An additional factor that might have a positive impact on the diversity of flower visitors is the proximity of protected forest to the fruit crops in San Gerardo, as has been previously shown for other tropical crops such as avocado, coffee, and mango (Carvalheiro et al., 2010; Celis-Diez et al., 2023; Hipólito et al., 2018; Villamil et al., 2018). Fruit crop farms in our study region are surrounded by ornamental gardens, stands of introduced cypress trees (*Cupressus lusitanica*), patches of secondary montane forest, and/or protected forest on the hilltops. The montane forests of San Gerardo are characterized by a species-rich plant community shaped in part by deforestation and other human activities since the mid 50’s. Clearance led to the emergence of open habitats such as pasture land and shrublands that enabled the migration and establishment of herbaceous plants from the higher altitude páramo and subalpine dwarf forests (Kappelle et al., 2000). It is possible that, besides providing additional feeding resources for pollinators, ruderal vegetation in and around fruit crops and undisturbed nearby montane forests provide microhabitats that sustain rich and abundant populations of insect pollinators. For instance, larvae of the dipteran family Sciaridae, feed on decaying plant material, an abundant substrate on the ground covered by the layer of ruderal plants and inside forest edges. Other larvae scavenge on dead bodies of wild (small and large) animals that die in the forest or feed on mammal dung (e.g., Sarcophagidae); yet the larvae of various fly families recorded in our study (e.g., Syrphidae) prey on the larvae of other insects (Rojo et al., 2003; Zumbado, 2006). The bumblebee *Bombus ephippiatus*, the most common native species in our study, builds nests underground, usually in drained terrain covered with a dense herbaceous layer (Laverty and Plowright, 1985); GB unpublished data).

Our study revealed that avocado farms tend to host a higher diversity of insect floral visitors compared to other fruit crops. The presence of a diverse floral visitor community is consistent with previous studies of avocado orchards (Okello et al., 2021; Perez-Balam et al., 2012; Villamil et al., 2018). We found that fruit crops and wild plants have a distinct community composition of flower visitors, and that insect composition was also specific to the type of fruit crop. Together, these results suggest that pronounced differences in microhabitat influence the presence or absence of insect taxa at different sampling sites. However, we are currently unable to distinguish the factors shaping these diversity patterns. Studies that incorporate surveys of wild plant diversity in parallel with insect surveys in farmlands might provide useful insights for future farming practices, such as evaluating the relationship between wild plant diversity and insect visitor diversity.

We found an effect of season on insect visitor community composition. Climate models predict that high-elevation tropical environments will experience a strong decrease in precipitation, higher temperatures, and increased variability of both precipitation and temperature (Karmalkar et al., 2008). Our results suggest that insect composition in San Gerardo are sensitive to seasonal effects. Given that high-elevation tropical ecosystems are regions of high endemism but also emerging hot-spots for climate change (Giorgi, 2006), long-term assessments of flower visitor community dynamics are crucial for understanding the potential effects of changing climatic conditions on plant-pollinator interactions.

Overall, the observed diversity of flower visitors in this study provides a baseline understanding of pollinator communities and their potential role as bioindicators of ecosystem health. The observed variability in diversity patterns of flower visitors across fruit crops and wild plant transects suggests the presence of fine-scale habitat differences that deserve further attention. For instance, the need to conduct studies that aim at identifying the main predictors of insect diversity and composition, such as the influence of distance to protected forest, the effect of farming practices, and the relationship between different wild plant assemblages and pollinator diversity. Currently, Latin America’s landscape conversion is largely driven by regional development (Keys, 2010; Klepeis and Turner, 2001; Newcomer et al., 2022). A main challenge to preserving biodiversity is therefore not only to effectively manage protected areas but also to ensure that rural areas develop in a more sustainable manner (Lambin et al., 2003). Costa Rica, despite its known efforts to protect biodiversity, represents a good example of this struggle (Broadbent et al., 2012; Kappelle and Juárez, 1995; Sanchez-Azofeifa et al., 2002). Our results suggest that tropical highland farmers have the potential to improve their crop yields and preserve ecosystem services by encouraging the growth of ruderals in and around their fields and by preserving tracts of undisturbed habitat.

### Diversity of plant sources revealed by pollen metabarcoding

A main outcome of this study was the relatively large number of plant species identified in pollen samples that were not necessarily observed in the fruit crops nor in the wild plant transects. We found that a high proportion of plant sources (close to 50%) were detected in up to two samples, illustrating the sensitivity of pollen metabarcoding in detecting rare taxa among flower visitors (Bell et al., 2017; Milla et al., 2021; Richardson et al., 2021). Insect visitors carried pollen from common ruderals and a variety of wild and endemic species. Some of these correspond to plant species with distributions restricted to the Talamanca mountain range (i.e., *Vaccinium consanguineum* (Ericaceae), *Ageratina kupperi* (Asteraceae), *Viburnum costaricanum* (Adoxaceae), *Hypericum irazuense* (Hypericaceae), *Sciodaphyllum pittieri* (Araliaceae), *Quercus costaricensis* (Fagaceae) and *Diplostephium costaricense* (Asteraceae)). These findings provide evidence that insect visitors benefit from the resources provided by vegetation co-occurring in fruit crop farms, plants from ornamental gardens, and nearby undisturbed habitat. This diverse community of flower visitors likely contributes to the pollination services of fruit crops and native plant species in San Gerardo, with nearby protected areas playing a crucial role in maintaining pollinator populations during periods when crop flowers are not in bloom.

Results of plant sources among pollen samples in fruit crops mirror the differences in composition observed in insect visitor data, i.e., the type of fruit crop was a strong predictor of similarity among pollen samples, suggesting that fruit crop farms have a relatively distinct community of herbaceous wild plants that attract different insect visitors and that these visitors exploit different foraging resources. Furthermore, we found that estimates of plant species diversity (Hill-evenness) in pollen samples were higher in apple and plum farms compared to avocado and blackberry crops. Differences in the distance between crops and the natural protected areas might contribute to the observed variation in diversity. The plum and one of the two apple farms were closer to the montane protected forest compared to the other study sites. Higher diversity in these fruit crops might reflect the greater availability and diversity of floral resources associated with nearby undisturbed habitats (Muñoz et al., 2021). The fact that the number of plant taxa detected per sample was highly variable suggests that a large proportion of plant sources identified through analyses of pollen samples correspond to the detection of heterospecific pollen picked up by flower visitors. High levels of heterospecific pollen can be an indication of high diversity of floral resources (Willmer et al., 2017) and phenological overlap (Aizen and Rovere, 2010).

We found differences in diversity with respect to groups of insect visitors; estimates of Hill-evenness were lower in syrphid flies compared to the other insect groups in our study. Syrphid flies appear to carry pollen of a few dominant plant species, including common ruderals planted by farmers to attract pollinators (e.g., pinkweed, *Persicaria capitata*). Lower diversity of plant sources in Syrphid flies might be explained by differences in morphological traits such as smaller body length and fewer setae compared to other flies like Tachinidae, which hinder pollen adherence (Cullen et al., 2021; Földesi et al., 2021).

Montane tropical forests are characterized by low temperatures and very humid conditions, which may have a negative effect on the activity of managed populations of honeybees (Corbet et al., 1993). In contrast, non-syrphid flies are more resilient to these unfavorable conditions (Levesque and Burger, 1982), which explains similar estimates of diversity of plant sources for non-syrphid flies compared to *Apis* and *Bombus* bees. Moreover, the high diversity of plant sources found in bumblebee samples highlights the importance of these insects as crop and wild plant pollinators. Bumblebees are the most prevalent wild pollinators in the understory of montane forests in the region (Cristóbal-Perez et al., 2024), remain active throughout the day, and have been shown to carry a high percentage of conspecific pollen grains (Wesselingh et al., 2000). Together, our findings echo previous work demonstrating the importance of studying wild pollinators in farmlands and their role in ecosystem services (Larson et al., 2001; Orford et al., 2015).

Our findings suggest that small-scale fruit crops may facilitate insect movement between forest patches, increasing gene flow and decreasing genetic structure among native and endemic plant species (Jha and Dick, 2010). Increased connectivity is crucial for maintaining the genetic diversity of native species, which is essential for their adaptability and population viability (Frankham, 2005; Newman and Tallmon, 2001; Richards, 2000). However, agricultural expansion may compromise landscape connectivity, resulting in habitat fragmentation that could disrupt insect movement between forest patches, reducing gene flow and thereby increasing the detrimental effects of genetic drift on native plant populations (Fuchs et al., 2003). Consequently, our results highlight the importance of preserving natural habitats and managing agricultural landscapes in a way that safeguards the genetic variability of native flora (Kamm et al., 2009; Van Geert et al., 2010).

Pollen metabarcoding is a valuable tool to assess the diversity of plant taxa available to insect visitors; however, this information is largely qualitative (Bell et al., 2019) and can be subject to amplification, sequencing and data processing biases (Arstingstall et al., 2021). While this method enabled us to characterize potential foraging resources associated with farmland near protected montane forests in Costa Rica, distinguishing pollination from other types of interactions (i.e., neutral or resource parasitism) remains a challenge. Future studies that combine direct measurements of pollinator effectiveness (e.g., via studies of single-visit deposition King et al., 2013) with pollen metabarcoding to identify plant sources in flower visitors are needed to evaluate the extent of pollination services provided by the diverse community of flower visitors observed in tropical montane agrosystems.

## Concluding remarks

The highland forests in Costa Rica are a species-rich region with very high levels of endemism (Horn and Kappelle, 2005; Anderson et al., 2008). Reliably assessing high species richness has been a major limitation to our understanding of plant-pollinator communities in the tropics (Freitas et al., 2009). Monthly surveys of insect flower visitors in farmlands located in the montane forests of San Gerardo revealed the presence of a diverse insect community. Through pollen metabarcoding techniques, we successfully examined the diversity of plant sources available for insect communities associated with fruit crops. These data revealed that insects visit a wide variety of native flowers, including endemic plants and plants found primarily in natural protected areas. These results highlight the important role of undisturbed habitat in sustaining pollinator populations. Furthermore, our findings indicate that a custom-built reference database that incorporates sequences from local plants is crucial for capturing genus or even species-level diversity, and provides empirical support for the value of pollen analyses to characterize and monitor local biodiversity (Bell et al., 2023; Hornick et al., 2022). Overall, this study emphasizes the interdependence of wild and cultivated plants, highlighting the importance of preserving natural habitats for the ecosystem services they provide.

## Data Availability

The data presented in the study are deposited in the https://figshare.com/ repository, accession number, DOI: 10.6084/m9.figshare.26021917. The scripts to reproduce all analyses and figures are available in the GitHub repository: https://github.com/bkmontero/PollenMetabarcodingUCR.
