# Supplementary material for: Pollen metabarcoding reveals a broad diversity of plant sources available to farmland flower visitors near tropical montane forest
Source: Front Plant Sci. 2025 Jan 7;15:1472066. doi: 10.3389/fpls.2024.1472066 (PMC11745891; doi:10.3389/fpls.2024.1472066)
Supplement: Supplementary file 1 [file Table1.pdf]

Supplementary material

Pollen metabarcoding reveals a broad diversity of plant sources available to farmland flower visitors near tropical montane forest

B. Karina Montero[1,2*], Nicole Gamboa-Barrantes[1,2], Geovanna Rojas-Malavasi[1,2], E. Jacob Cristóbal-Perez[4,5], Gilbert Barrantes[1,2,5], Alfredo Cascante-Marín[1,2,5], Paul Hanson[1,2], Manuel A. Zumbado[7], Ruth Madrigal-Brenes[1,2,5], Silvana Martén-Rodríguez[4,5,6], Mauricio Quesada[4,5] and E. J. Fuchs[1,2,3,4]

[1]*Escuela de Biología, Universidad de Costa Rica, San José, Costa Rica*
[2]*Centro de Investigación en Biodiversidad y Ecología Tropical, Universidad de Costa Rica, San José, Costa Rica*
[3]*Laboratorio Nacional de Análisis y Síntesis Ecológica, Escuela Nacional de Estudios Superiores, Unidad Morelia, Universidad Nacional Autónoma de México, Morelia, Michoacán, México*
[4]*Laboratorio Binacional de Análisis y Síntesis Ecológica, UNAM-UCR, México-Costa Rica*
[5]*Laboratorio de Ecología Evolutiva de Plantas, Escuela Nacional de Estudios Superiores-Morelia, Universidad Nacional Autónoma de México, Morelia, Michoacán, México*
[6]*Investigador Colaborador, Museo de Zoología, Universidad de Costa Rica, San José, Costa Rica*
[*]*Present address: Biodiversity Research Institute (CSIC, Oviedo University, Principality of Asturias), Campus of Mieres, University of Oviedo, Mieres, Spain*

Supplementary Text S1

Sequencing methods for building a local plant database

We collected and sequenced endemics and common plant species (native and naturalized) in the region of San Gerardo and the neighbouring páramo habitat to build a database for taxonomic classification of sequence variants that accounted for the local diversity of the study site. For this purpose, we collected ca 100 g of leaf tissue and immediately stored it in a falcon tube (50 ml) with silica powder. Vouchers of all plant species are stored in the Herbarium of the University of Costa Rica. We took samples to the Molecular Ecology Laboratory at the Universidad de Costa Rica, where silica was renewed at least twice to ensure that plants were adequately dry before DNA extraction. We extracted DNA from 140 species using the modified CTAB protocol proposed by Doyle and Doyle (1987). We used approximately 50 grams of dry tissue that was previously cut into tiny pieces using a scalpel. Tissue was pulverized using liquid nitrogen in a mortar and transferred to a 1.5 ml tube; 500 uL of warm (50°C) CTAB buffer was immediately added to the pulverized sample. We then followed the standard protocol. DNA quantity and quality was assessed using a Nanodrop. The 260/280 and 230/260 ratios were checked for all samples; those samples that diverged from expectations were extracted again. We amplified a 400-450bp ITS2 fragment using the primers by Cheng et al. (2016). Amplification reactions were performed in a total volume of 25 ul with 50 ng of template DNA, 0.2 uM of forward and reverse primers and 12.5 ul of GoTaq® Colorless Master Mix 2x (Promega). The PCR was carried out using a Verity thermocycler (Applied Biosystems). The cycling program consisted of the following steps: 5 min at 94°C followed by 30 cycles of 90s at 55°C and 90s at 72°C and a final extension step of 72°C for 10min. Sequencing was performed in both directions at Macrogene. We trimmed sequence tips with error rates higher than 4% and assembled forward and reverse sequences using the default Geneious algorithm, in Geneious Prime® 2022.2.1. In addition, we checked by hand the contigs to resolve discrepancies, and extracted consensus sequences for downstream analyses.

Custom-built database strategies

To compare the influence of sequence processing-steps used to create custom-built databases on taxonomic assignment of sequence variants, we tested the following combination of parameters: i. dereplication (i.e. derep), ii. extraction of primer specific amplicon-region (i.e. primer trimmed), and iii. geographic restriction of retrieval of NCBI sequences. We used the rescript plugin in qiime2 to dereplicate identical sequences in the reference sequence data using the option *-uniq* (Robeson II et al., 2021). We used the module *crabs pga* from the software package CRABS (Creating Reference databases for Amplicon-Based Sequencing) (Jeunen et al., 2023) to extract the amplicon-region from the reference database using the ITS2 primers (Cheng et al., 2016). Finally, we generated a global database with no geographic restriction and a reference database with geographic restriction to Mexico, Central and South America, and the Caribbean. For this step, the ITS2 marker sequences published in the NCBI database were downloaded using the "Entrez query" *((viridiplantae[Organism] AND its2) AND 100:10000000[Sequence Length]) NOT (uncultured OR environmental sample OR incertae sedis OR unverified)*, and ((viridiplantae[Organism] AND its2) AND 100:10000000[Sequence Length] AND (Mexico OR Guatemala OR Belize OR El Salvador OR Belize OR Honduras OR

Nicaragua OR Costa Rica OR Panama OR Cuba OR Jamaica OR Haiti OR Dominican Republic OR Caribbean OR Puerto Rico OR Colombia OR Venezuela OR Guyana OR Suriname OR French Guyana OR Ecuador OR Peru OR Brazil OR Bolivia OR Paraguay)) NOT (uncultured OR environmental sample OR incertae sedis OR unverified) on March 3, 2023. In addition, we were interested in testing to what extent merging (or not) a local database with the NCBI reference database impacted the taxonomic assignment of sequence variants. In total, this resulted in 16 databases (and classifiers).

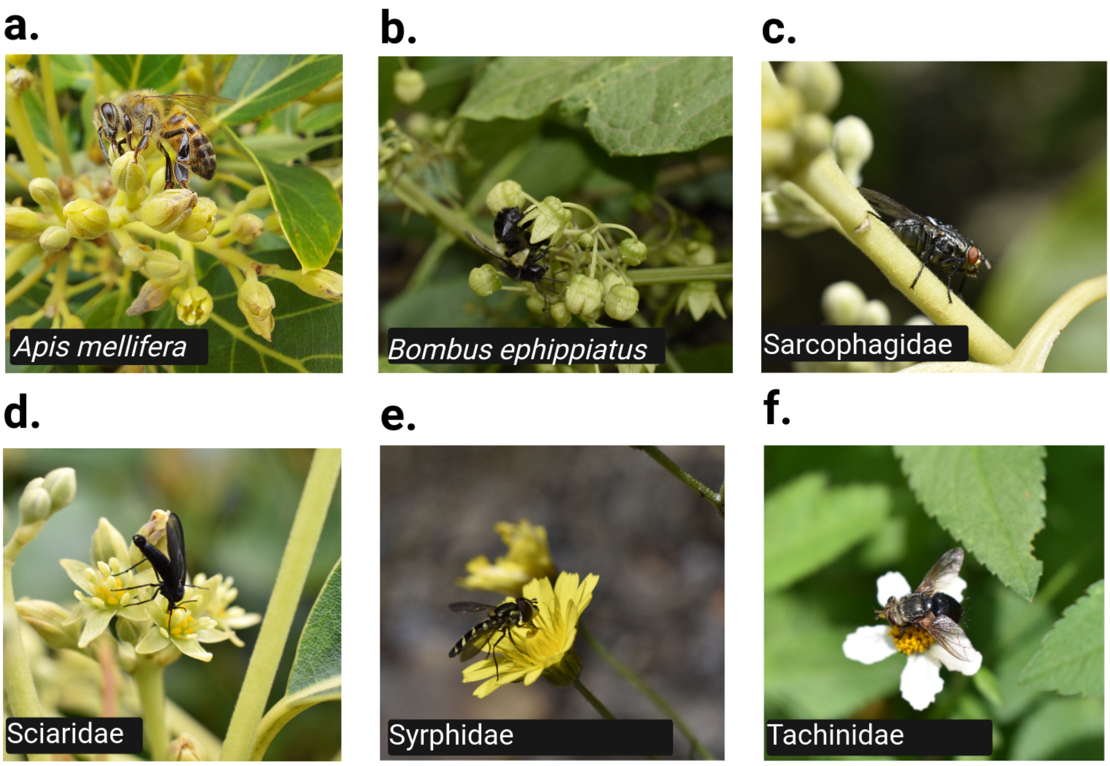

Supplementary Figure 1: Representative insect visitors in San Gerardo. a-b) Hymenoptera. c-f) Diptera. Photo credits: Geovanna Rojas-Malavasi.

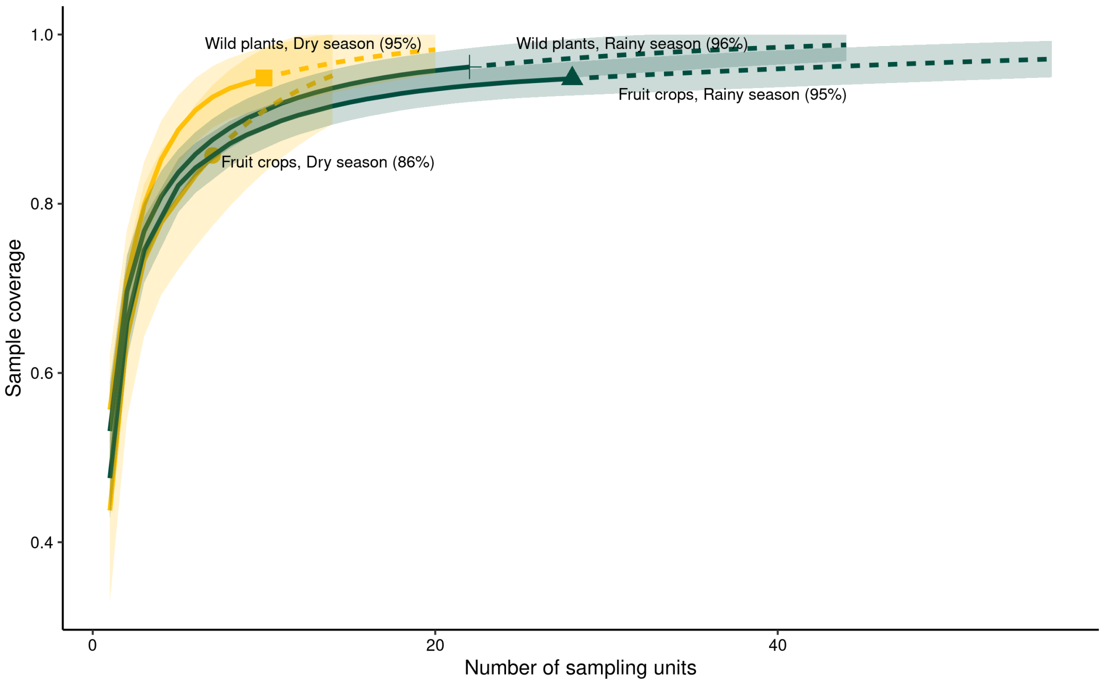

Supplementary Figure 2: Sample completeness curves of fruit crops and wild plant transects for the dry and the rainy season in San Gerardo.

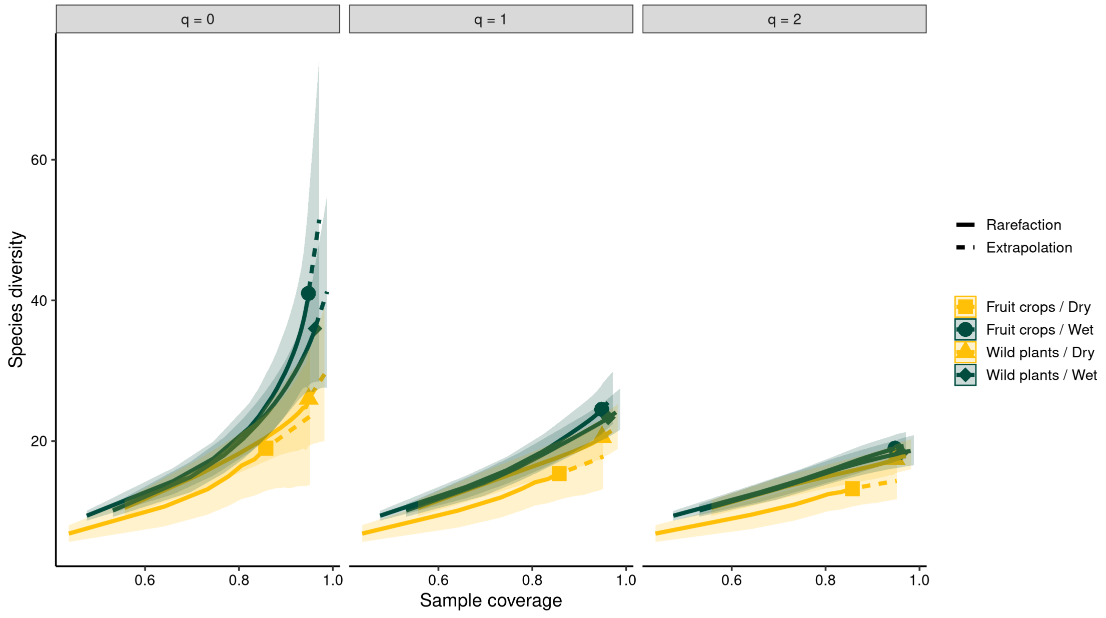

Supplementary Figure 3: Coverage-based rarefaction (solid line) and extrapolation (dotted line) curves for insect visitors sampled in fruit crops and wild plant transects during the dry (yellow) and rainy (green). Diversity estimates are separated by panels, richness (q = 0), Hill-Shannon (q = 1), and Hill-Simpson (q = 2).

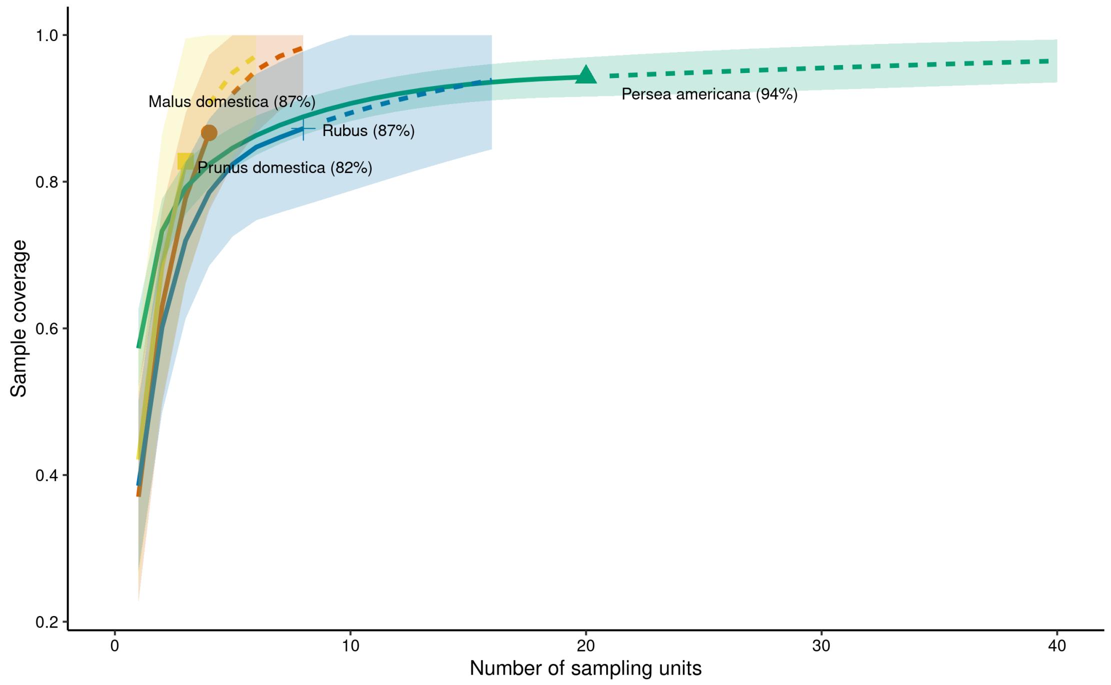

Supplementary Figure 4: Sample completeness curves of fruit crops in San Gerardo.

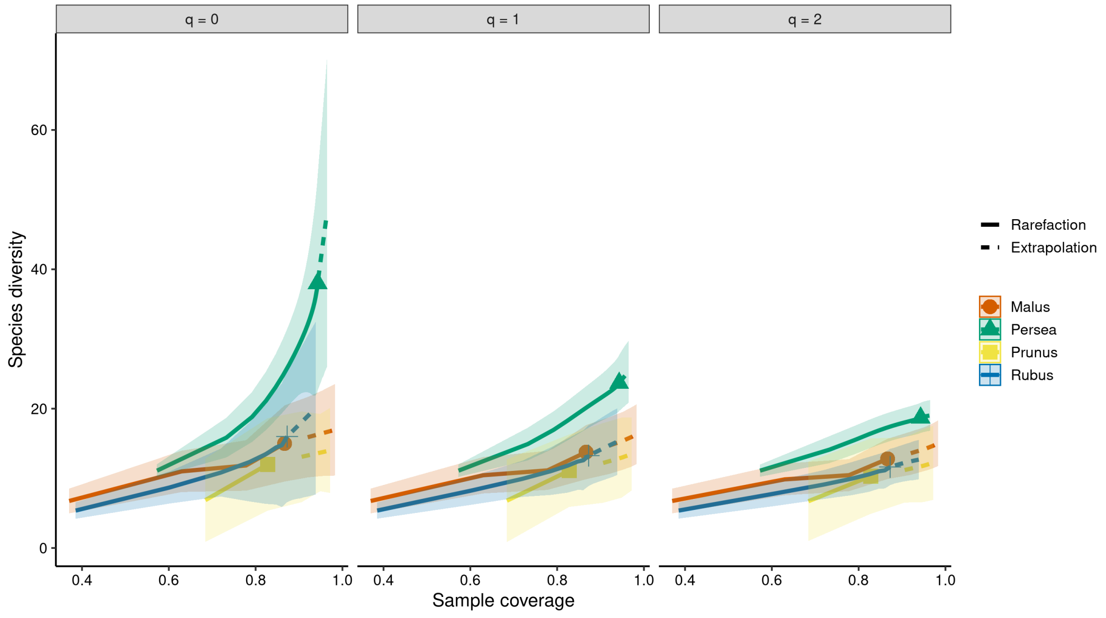

Supplementary Figure 5: Coverage-based rarefaction (solid line) and extrapolation (dotted line) curves for insect visitors sampled in apple (*Malus domestica*), avocado (*Persea americana*), plum (*Prunus domestica*, and blackberry (*Rubus* spp.). Diversity estimates are separated by panels, richness (q = 0), Hill-Shannon (q = 1), and Hill-Simpson (q = 2).

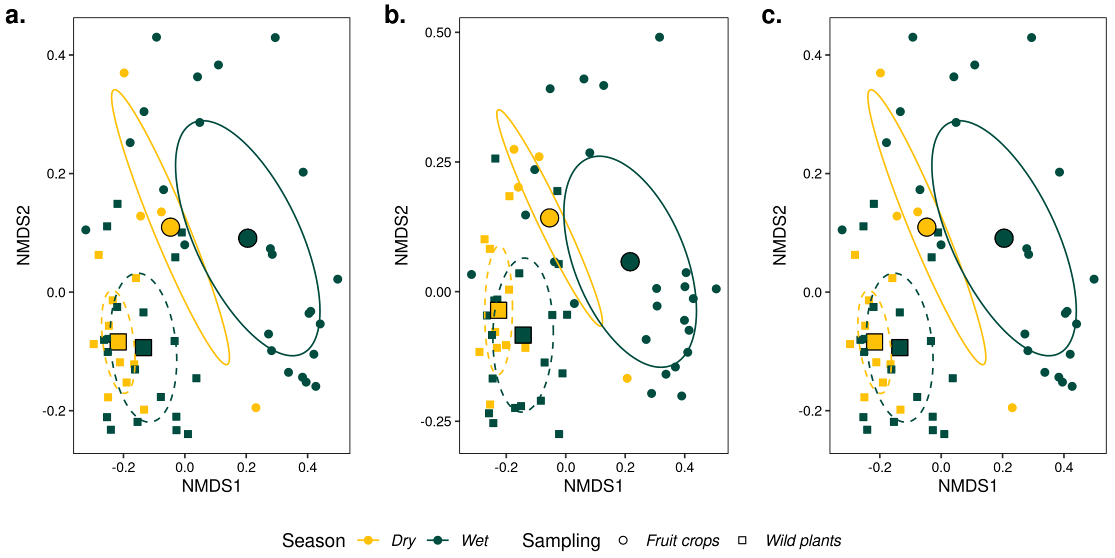

Supplementary Figure 6: Non-metric multidimensional scaling (NMDS) of insect visitor composition using the Hill number Sørensen-type dissimilarity across three q levels (a. q = 0, stress = 0.121; b. q = 1, stress = 0.126 ; c. q = 2, stress = 0.121). Samples from fruit crops correspond to filled dots whilst samples from wild plants are shown with filled squares. Seasons are designated by colour (yellow for dry season and green for wet season). Shaded ellipses denote the standard error of the centroid insect community for each sampling and season at 95% confidence. Solid lines correspond to insect visitors from fruit crops and dotted lines from wild plants.

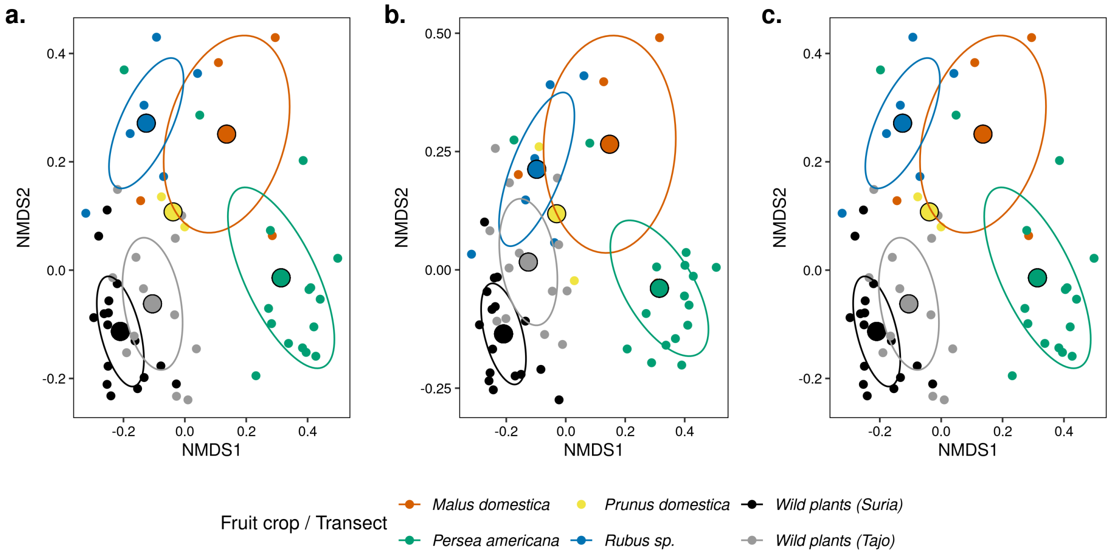

Supplementary Figure 7: Non-metric multidimensional scaling (NMDS) illustrating the differences in insect visitor composition across 4 fruit crops and 2 transects of wild plants in San Gerardo. The analysis was done using the Hill number Sørensen-type dissimilarity across three q levels (a. q = 0, stress = 0.121; b. q = 1, stress = 0.126 ; c. q = 2, stress = 0.121). Shaded ellipses denote the standard error of the centroid insect community for each fruit crop and transect at 95% confidence. Note that for plum, sample size is insufficient to draw an ellipse (N sampling units = 2).

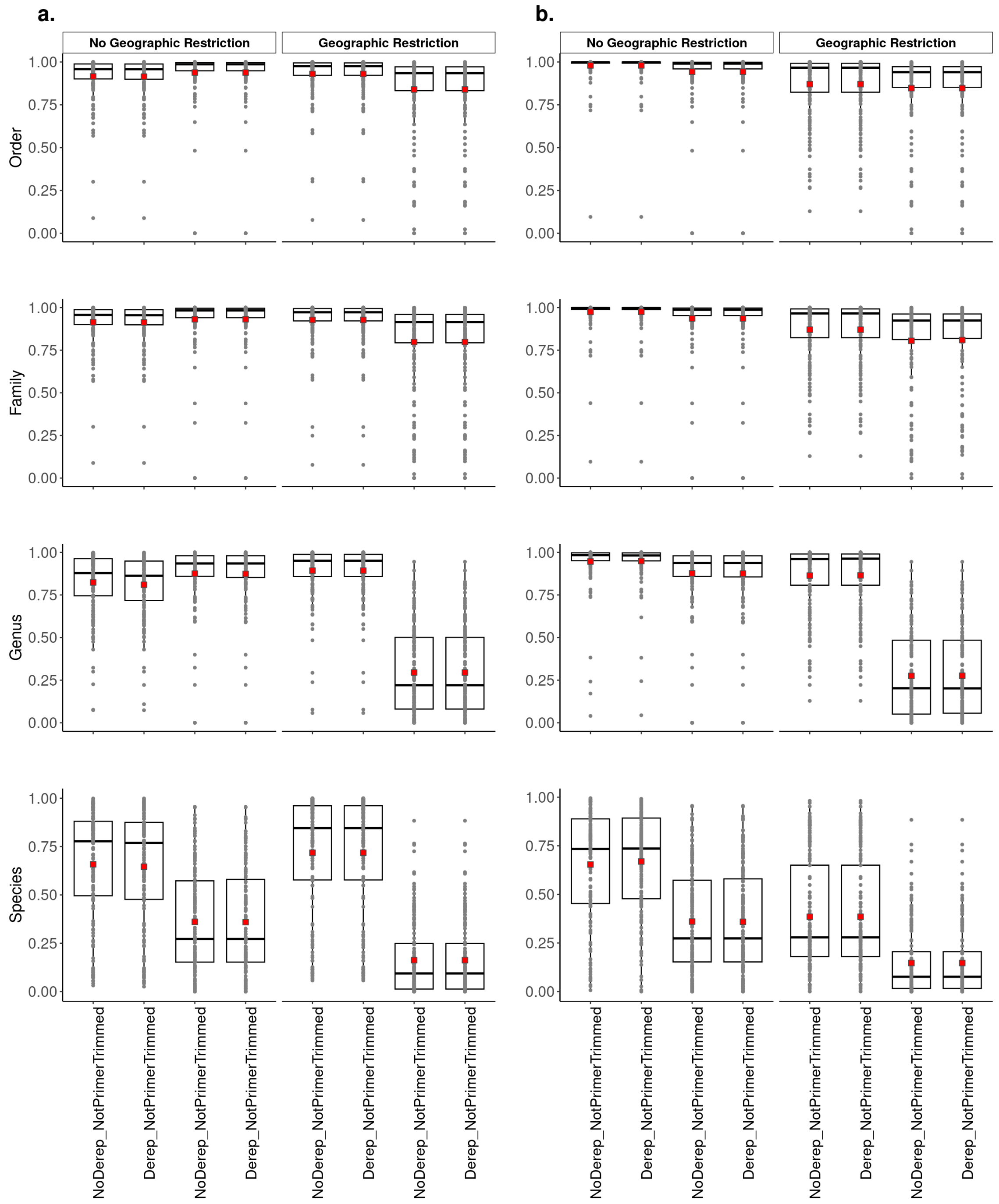

Supplementary Figure 8: Fraction of reads assigned to order, family, genus and species using custom-built reference databases with different sequence processing parameters (i.e. dereplication and primer trimmed extracted amplicons) and NCBI sequence retrieval strategies (i.e. with and without geographic restriction). (a.) NCBI database merged with local database. (b.) NCBI database. Assignment above Order level was 100% across all databases.

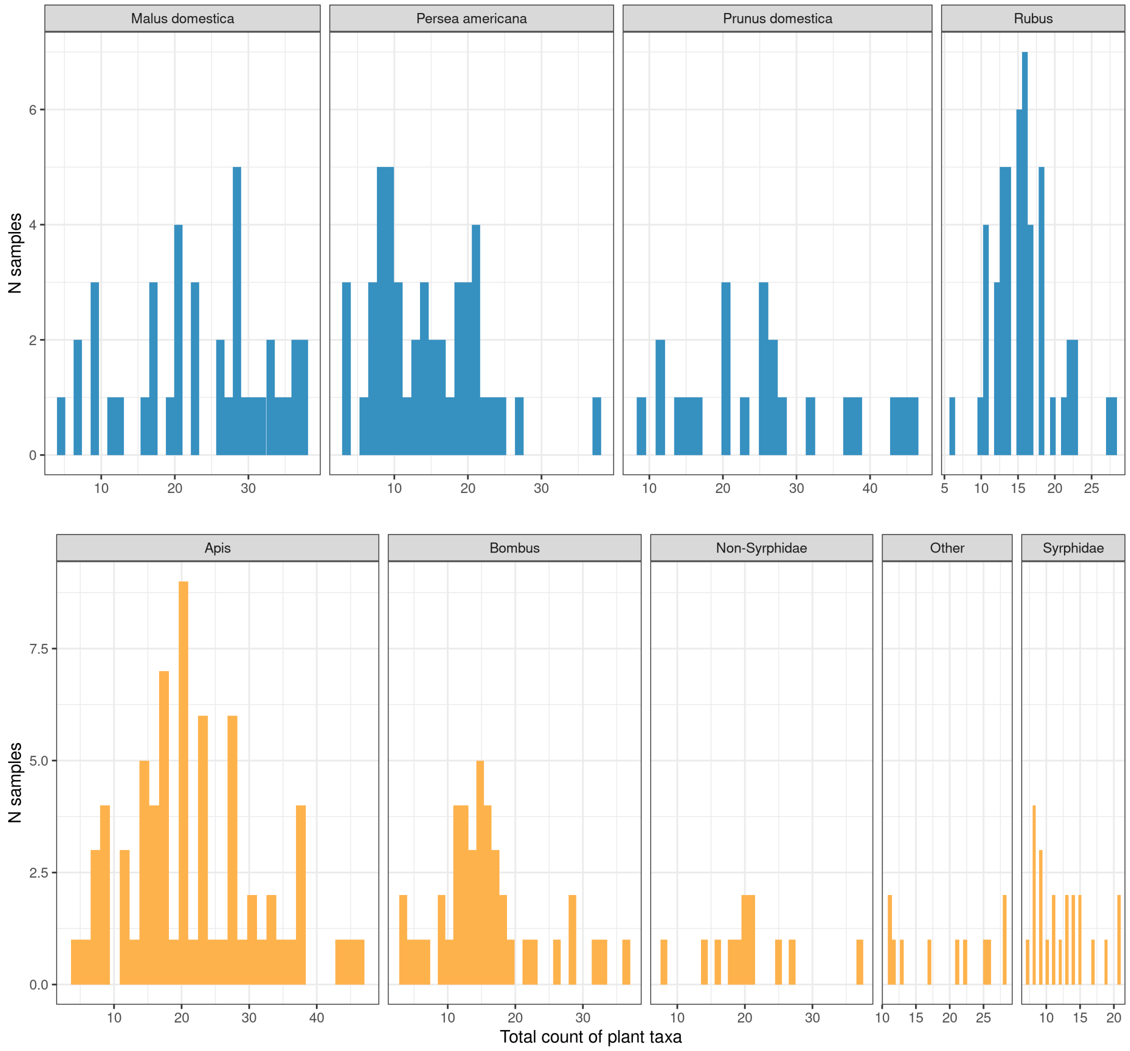

Supplementary Figure 9: Histograms showing the total count of plant species (or genera) detected across samples. Samples are grouped by fruit crop (upper panel) and by insect group (lower panel).

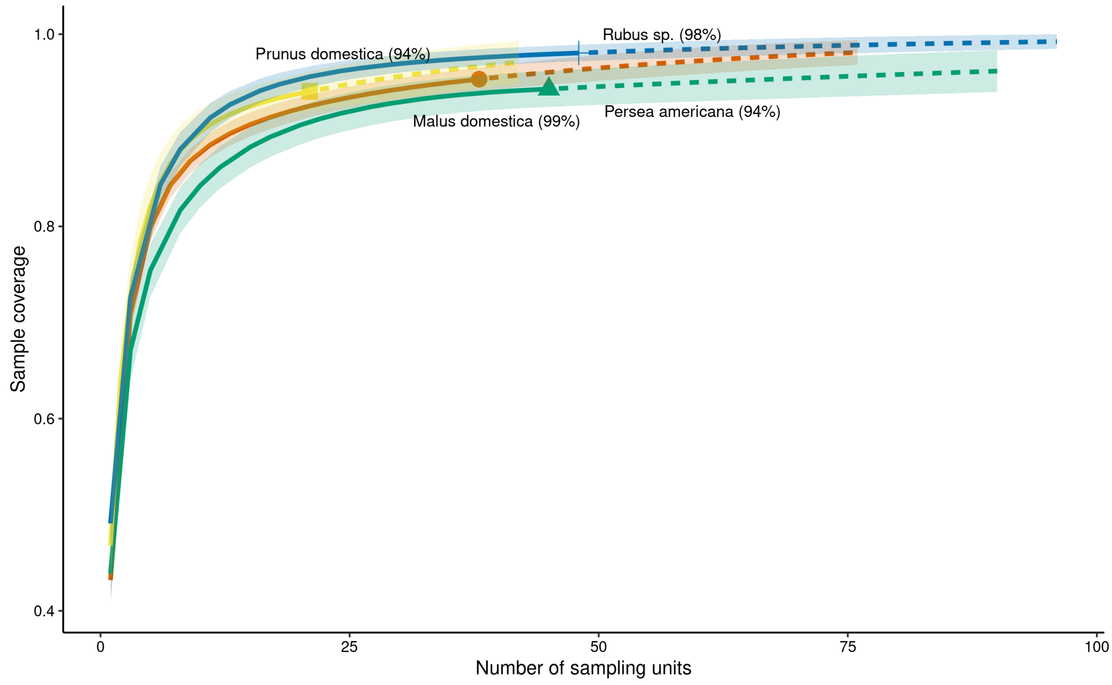

Supplementary Figure 10: Sample completeness curves of plant taxa identified from pollen samples of insect visitors collected in four fruit crops San Gerardo.

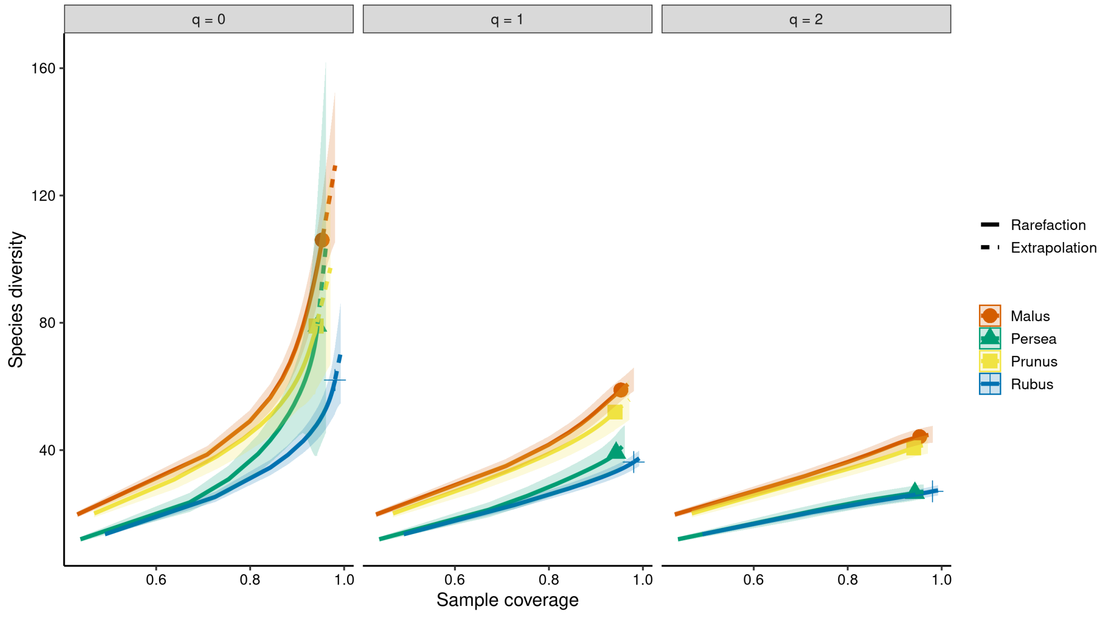

Supplementary Figure 11: Coverage-based rarefaction (solid line) and extrapolation (dotted line) curves for plant diversity estimated from pollen samples collected from insect visitors grouped by fruit crops. Diversity estimates are separated by panels, richness (q = 0), Hill-Shannon (q = 1), and Hill-Simpson (q = 2).

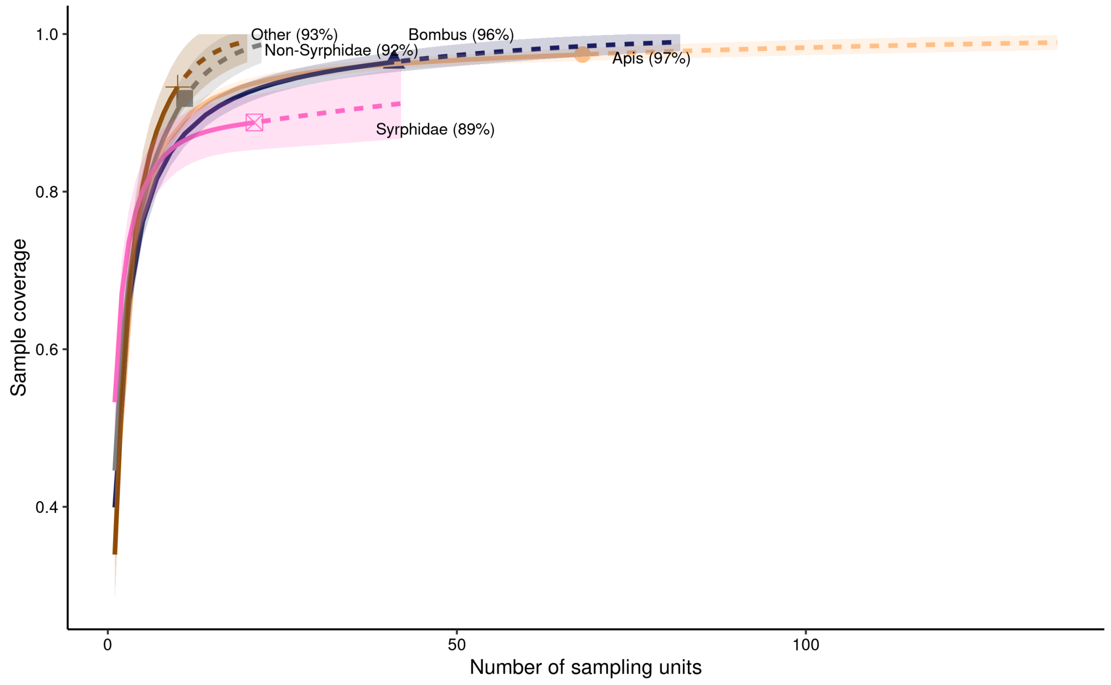

Supplementary Figure 12: Sample completeness curves of plant taxa identified from pollen samples according to insect visitors in San Gerardo.

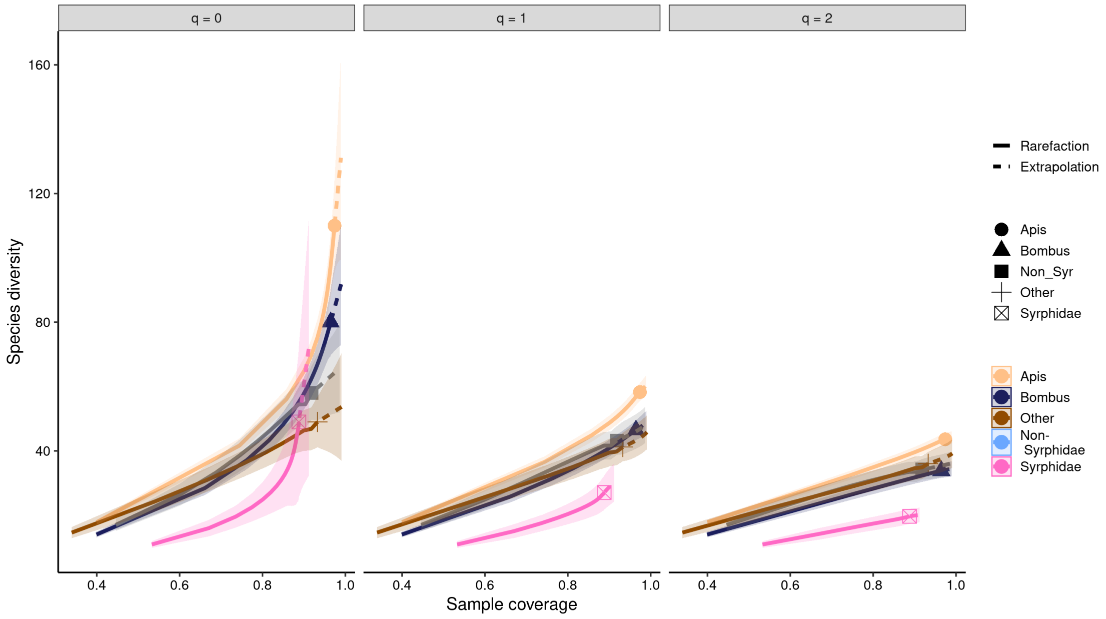

Supplementary Figure 13: Coverage-based rarefaction (solid line) and extrapolation (dotted line) curves for plant diversity estimated from pollen samples across insect visitors. Diversity estimates are separated by panels, richness (q = 0), Hill-Shannon (q = 1), and Hill-Simpson (q = 2).

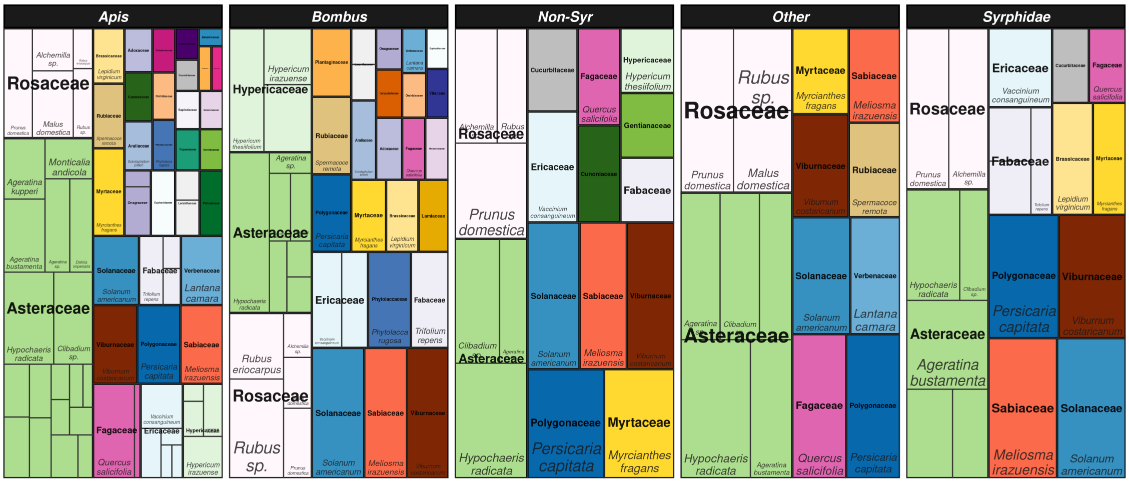

Supplementary Figure 14: Treemap showing the composition of plant taxa (>3% within insect group prevalence) found across insect groups. Each tile represents a plant species (or genus), with labels shown for common species only (>20%). The area of the tile and label font size is proportional to the within fruit crop prevalence. The fill colors correspond to plant families.

Supplementary Table 1: List of wild herbaceous and shrub-like plant species surveyed in San Gerardo from 2021 to 2022. Numbers correspond to the number of insect surveys (i.e. sampling sessions) conducted for a particular species on a given month, if it was flowering. A subset of wild plants were common ruderal species found in the fruit crop farms ("Ruderal"), noted here as "yes".

**Family**	**Species**	**Jan**	**Feb**	**Mar**	**Apr**	**May**	**Jun**	**Jul**	**Aug**	**Sep**	**Oct**	**Nov**	**Dec**	**Ruderal**
Acanthaceae	*Thunbergia alata*	0	0	0	1	0	0	0	0	0	1	0	1	no
Asteraceae	*Ageratina bustamenta*	1	2	1	0	0	0	0	0	0	0	0	2	no
	Ageratum conyzoides	1	1	0	0	0	0	1	0	1	2	1	2	no
	Bidens pilosa	1	0	0	1	0	0	3	2	3	3	2	2	yes
	Bidens reptans	2	1	0	0	1	1	1	0	0	0	2	1	no
	Clibadium leiocarpum	0	0	0	0	0	0	0	2	2	2	1	1	no
	Conyza sumatrensis	0	0	0	0	0	1	1	2	0	0	0	0	yes
	Crepis capillaris	1	2	2	2	2	1	2	2	3	2	1	1	yes
	Dahlia imperialis	0	0	0	0	0	0	0	0	1	1	1	1	yes
	Hypochaeris radicata	1	1	2	2	1	1	2	3	0	3	2	2	yes
	Jaegeria hirta	0	0	0	0	0	0	0	0	0	1	0	2	yes
	Leucanthemum vulgare	0	0	0	0	0	0	0	0	0	0	1	1	no
	Pseudognaphalium attenuatum	0	1	1	0	0	0	0	0	0	0	0	0	no
	Sonchus oleraceus	0	0	1	1	0	1	2	0	1	1	1	0	yes
	Viguiera cordata	0	1	1	0	0	0	0	0	0	0	1	0	no
Balsaminaceae	*Impatiens sodenii*	1	0	0	0	0	0	0	0	0	1	0	1	yes
Boraginaceae	*Wigandia urens*	1	1	1	0	0	0	0	0	0	1	0	1	no
Brassicaceae	*Lepidium virginicum*	0	0	0	0	1	1	1	1	0	0	0	0	yes
Caryophyllaceae	*Arenaria lanuginosa*	0	0	0	0	0	0	1	0	0	0	0	0	yes
Convolvulaceae	*Ipomoea purpurea*	1	0	1	1	1	1	2	1	1	2	1	1	no
Cucurbitaceae	*Cyclanthera langaei*	0	0	0	0	0	0	0	0	1	0	0	0	no
	Sechium pittieri	1	1	1	0	1	1	2	1	2	2	1	0	yes
Fabaceae	*Phaseolus dumosus*	0	0	0	0	1	1	0	1	1	1	1	1	no
	Senna guatemalensis	1	0	1	1	0	0	0	0	0	0	0	0	no
	Trifolium repens	1	0	1	0	0	0	1	1	1	1	0	0	yes
Geraniaceae	*Geranium seemannii*	1	0	0	0	1	0	0	0	2	2	1	1	no
Hypericaceae	*Hypericum thesiifolium*	0	0	0	0	0	0	1	0	0	0	0	0	yes
Melastomataceae	*Monochaetum floribundum*	0	0	0	0	0	0	0	1	0	0	0	0	no
Onagraceae	*Fuchsia microphylla*	0	0	1	0	0	0	0	0	0	0	0	0	no
	Fuchsia paniculata	0	0	0	0	0	0	0	0	0	1	1	0	no
	Lopezia miniata	2	2	1	1	0	0	1	1	1	1	1	2	no
Passifloraceae	*Passiflora ligularis*	0	1	0	0	1	1	1	0	0	2	0	1	no
Phrymaceae	*Hemichaena fruticosa*	1	0	0	0	0	0	0	0	0	0	0	1	no
Phytolaccaceae	*Phytolacca rugosa*	0	0	0	1	0	0	0	0	0	0	0	0	no
Polygonaceae	*Muehlenbeckia tamnifolia*	1	0	0	0	0	1	3	2	2	2	1	1	no
	Persicaria capitata	1	2	1	2	2	2	3	2	3	1	2	2	yes
	Rumex obtusifolius	0	0	0	0	1	0	1	0	0	1	0	0	yes
Rosaceae	*Rubus adenotrichus*	0	0	0	1	0	0	1	0	0	0	0	0	yes
	Rubus costaricanum	1	1	0	0	0	0	0	0	0	0	0	1	no
Rubiaceae	*Spermacoce remota*	0	0	0	0	0	2	3	2	0	0	0	0	yes
Scrophulariaceae	*Veronica serpyllifolia*	0	0	0	1	1	1	0	0	0	0	0	0	yes
Solanaceae	*Brugmansia arborea*	1	1	0	1	0	0	0	1	1	1	1	1	yes
	Solanum aligerum	0	0	0	0	0	1	0	0	0	0	0	0	no
	Solanum americanum	0	0	0	0	1	0	1	1	0	0	0	0	yes
Verbena litoralis	*Verbena litoralis*	1	0	2	2	2	2	3	3	1	2	1	1	yes
Vitaceae	*Cissus obliqua*	0	0	0	0	1	0	0	0	0	0	0	0	no

Supplementary Table 2: Number of insect surveys (i.e. sampling sessions) conducted in San Gerardo from 2021 to 2022 in 4 fruit crops (avocado *Persea americana*, blackberry *Rubus sp.*, apple *Malus domestica*, and plum *Prunus domestica*) and 2 transects labelled "Suria" and "Tajo" where insect visitors found in local wild plants were surveyed.

**Year**	**Fruit crop/Transect**	**Jan**	**Feb**	**Mar**	**Apr**	**May**	**Jun**	**Jul**	**Aug**	**Sep**	**Oct**	**Nov**	**Dec**
2021	*Persea americana*	0	0	0	0	0	0	0	3	2	0	0	0
2022	*Persea americana*	1	1	0	0	0	0	5	4	0	0	0	0
2022	*Rubus* sp.	0	0	0	0	1	1	0	1	0	2	1	0
2021	*Malus domestica*	0	0	0	0	0	0	1	0	0	0	0	0
2022	*Malus domestica*	1	0	0	0	0	0	1	1	0	0	0	0
2021	*Prunus domestica*	0	0	0	0	0	0	1	0	0	0	0	0
2022	*Prunus domestica*	1	0	0	0	0	1	0	0	0	0	0	0
2021	Transect Suria	0	0	0	0	0	1	1	1	1	1	1	1
2022	Transect Suria	1	1	1	1	1	1	2	1	1	1	0	0
2021	Transect Tajo	0	0	0	0	0	1	0	0	1	1	1	1
2022	Transect Tajo	1	1	1	1	1	1	1	1	1	0	0	0

Supplementary Table 3: List of flower visitors in fruit crops and wild plants belonging to the Order Coleoptera.

Order	Family	Genus	Species/Morphospecies	Overall abundance
Coleoptera	Cantharidae	Chauliognathus	2	3
	Cerambycidae	Scatopyrodes	1	2
	Chrysomelidae	Brachypnoea	1	1
	Chrysomelidae		0	6
	Coccinellidae		0	3
	Curculionidae		0	4
	Elateridae		0	2
	Lampyridae		0	1
	Latridiidae		0	1
	Melyridae	Astylus	1	113
	Mycteridae		0	76
	Nitidulidae	Conotelus	1	25
	Nitidulidae		0	2
	Staphylinidae		0	3

Supplementary Table 4: List of flower visitors in fruit crops and wild plants belonging to the Order Diptera.

Order	Family	Genus	Species/Morphospecies	Overall abundance
Diptera	Agromyzidae		0	6
	Anisopodidae		0	2
	Anthomyiidae	Anthomya	1	1
	Anthomyiidae	Calythea	1	19
	Anthomyiidae	Eutrichota	1	8
	Anthomyiidae	Hydrophoria	1	13
	Anthomyiidae	Lasiomma	1	9
	Bibionidae		0	2
	Blephariceridae		0	1
	Bombyliidae	Dipalta	1	5
	Bombyliidae	Exoprosopa	1	1
	Bombyliidae	Paravilla	1	1
	Braconidae		0	1
	Calliphoridae	Calliphora	1	3
	Calliphoridae	Chrysomyia	2	3
	Calliphoridae	Compsomyiops	1	42
	Calliphoridae	Lucilia	2	51
	Ceratopogonidae		0	32
	Chironomidae		0	22
	Chloropidae	Agrophaspidium	1	1
	Chloropidae	Biorbitella	4	9
	Chloropidae	Fiebrigella	1	1
	Chloropidae	Malloewia	3	67
	Chloropidae	Olcella	2	65
	Chloropidae	Onychaspidium	3	13
	Chloropidae	Thaumatomyia	1	62
	Chloropidae	Tricimba	1	1
	Chloropidae		0	1
	Conopidae		0	4
	Culicidae		0	4
	Dolichopodidae		0	4
	Drosophilidae	Cladochaeta	1	2
	Drosophilidae	Drosophila	7	111
	Drosophilidae	Scaptomyza	2	12
	Drosophilidae	Zygothrica	1	6
	Empididae	Lamprempis	1	2
	Empididae	Porphyrochroa	1	5
	Empididae	Rhamphomyia	3	5
	Ephydridae		0	9
	Fanniidae	Fannia	1	1
	Heleomyzidae		0	8
	Hybotidae	Platypalpus	2	3
	Lauxaniidae		0	19
	Lonchaeidae		0	20
	Milichiidae	Milichella	1	3
	Muscidae	Brontaea	5	9
	Muscidae	Coenosia	2	6
	Muscidae	Heli	6	10
	Muscidae	Hydrotaea	3	13
	Muscidae	Limnophora	4	140
	Muscidae	Lispoides	2	13
	Muscidae	Micropotamia	1	1
	Muscidae	Morellia	1	3
	Muscidae	Mydaea	1	6
	Muscidae	Myospila	1	5
	Muscidae	Neodexiopsis	11	145
	Muscidae	Phaonia	2	3
	Muscidae	Pilispi	1	1
	Muscidae	Sarcopromusca	1	1
	Muscidae	Spilogo	1	1
	Muscidae	Stomoxys	1	1
	Muscidae		0	7

Supplementary Table 5

Order	Family	Genus	Species/Morphospecies	Overall abundance
Diptera	Mycetophilidae		0	13
	Periscelididae		0	1
	Phoridae		0	17
	Pseudopomyzidae	Pseudopomyza (Rhinopomyzella)	1	3
	Psychodidae		0	1
	Pteromalidae		0	1
	Sarcophagidae		0	148
	Scatopsidae		0	61
	Sciaridae		0	130
	Sepsidae		0	13
	Simuliidae		0	5
	Sphaeroceridae		0	2
	Stratiomyidae		0	1
	Syrphidae	Allograpta	10	245
	Syrphidae	Argentinomyia	5	49
	Syrphidae	Copestylum	1	1
	Syrphidae	Leucopodella	1	1
	Syrphidae	Ocyptamus	9	86
	Syrphidae	Palpada	5	28
	Syrphidae	Platycheirus	1	57
	Syrphidae	Quichua	3	12
	Syrphidae	Salpingogaster	1	1
	Syrphidae	Toxomerus	4	173
	Syrphidae		0	2
	Tachinidae	Acrocantha	1	1
	Tachinidae	Anisia	1	2
	Tachinidae	Archytas	1	2
	Tachinidae	Bombyliomyia	1	4
	Tachinidae	Calolydella	3	4
	Tachinidae	Ceromyia	1	1
	Tachinidae	Chaetogaedia	1	1
	Tachinidae	Chaetostigmoptera	4	17
	Tachinidae	Chrysoexorista	1	1
	Tachinidae	Clausicella	1	1
	Tachinidae	Disticho	1	1
	Tachinidae	Dolichotarsus	1	1
	Tachinidae	Epalpus	6	13
	Tachinidae	Erythromela	1	2
	Tachinidae	Eucelatoria	1	1
	Tachinidae	Eulobomyia	1	1
	Tachinidae	Gaediopsis	2	46
	Tachinidae	Ginglymyia	1	8
	Tachinidae	Hemisturmia	1	2
	Tachinidae	Jurinella	1	11
	Tachinidae	Leskia	3	19
	Tachinidae	Myiomima	3	15
	Tachinidae	Myiopharus	1	1
	Tachinidae	Mystacella	1	1
	Tachinidae	Neobrachelia	1	1
	Tachinidae	Neosolieria	1	1
	Tachinidae	Paradejeania	1	1
	Tachinidae	Parepalpus	4	12
	Tachinidae	Peleteria	3	7
	Tachinidae	Periscepsia	1	2
	Tachinidae	Phasia	1	3
	Tachinidae	Phasiophyto	1	1
	Tachinidae	Phytomyptera	3	6
	Tachinidae	Protodejeania	2	12
	Tachinidae	Pseudosipho	5	12
	Tachinidae	Ptilodexia	1	3
	Tachinidae	Scotiptera	1	1
	Tachinidae	Sipho	3	16
	Tachinidae	Sphaeri	1	1
	Tachinidae	Thelyoxynops	1	1
	Tachinidae	Trafoia	2	22
	Tachinidae	Trichophora	2	4
	Tachinidae	Xanthoepalpus	1	1
	Tachinidae		0	7
	Tanypezidae		0	1
	Tephritidae		0	8
	Tipulidae		0	6
	Ulidiidae		0	2

Supplementary Table 6: List of flower visitors in fruit crops and wild plants belonging to the Order Hemiptera.

Order	Family	Genus	Species / Morphospecies	Overall Abundance
Hemiptera	Anthocoridae		0	1
	Aphididae		0	13
	Cicadellidae		0	7
	Cixiidae		0	1
	Coniopterygidae		0	1
	Delphacidae		0	1
	Lygaeidae		0	1
	Miridae		0	9
	Rhyparochromidae		0	2
	Triozidae		0	1

Supplementary Table 7: List of flower visitors in fruit crops and wild plants belonging to the Order Hymenoptera.

Order	Family	Genus	Species/Morphospecies	Overall abundance
Hymenoptera	Apidae	Apis	1	695
	Apidae	Bombus	3	175
	Apidae	Cerati	5	11
	Apidae	Exomalopsis	1	9
	Apidae	Meliwillea	1	40
	Apidae	Partamo	1	15
	Apidae	Thygater	1	4
	Apidae		0	1
	Braconidae	Bracon	1	2
	Braconidae	Opius	1	3
	Braconidae		0	18
	Colletidae	Colletes	1	1
	Colletidae	Hylaeus	1	1
	Crabronidae	Ectemnius	2	2
	Crabronidae	Solierella	1	1
	Cynipidae		0	1
	Diapriidae		0	1
	Encyrtidae		0	3
	Eulophidae	Galeopsomyia	1	1
	Eulophidae		0	3
	Figitidae	Acharis	1	3
	Figitidae		0	22
	Formicidae	Tapinoma	1	1
	Formicidae		0	4
	Halictidae	Habralictus	1	2
	Halictidae	Lasioglossum	12	182
	Halictidae	Neocorynura	3	6
	Halictidae	Pereirapis	1	1
	Ichneumonidae	Dreisbachia	1	2
	Ichneumonidae		0	24
	Megachilidae	Megachile	1	1
	Pirenidae		0	1
	Platygastridae		0	4
	Proctotrupidae		0	3
	Pteromalidae	Lyrcus	1	4
	Pteromalidae		0	8
	Scoliidae		0	1
	Sphecidae	Podalonia	1	1
	Tenthredinidae		0	1
	Tiphiidae	Tiphia	3	19
	Tiphiidae		0	1
	Torymidae	Torymus	1	1
	Torymidae		0	1
	Vespidae	Agelaia	4	13
	Vespidae	Ancistrocerus	1	1
	Vespidae	Epipo	1	16
	Vespidae	Polybia	1	6
	Vespidae	c.f. Ancistrocerus	1	1
	Vespidae	c.f. Zeta	1	2

Supplementary Table 8: List of flower visitors in fruit crops and wild plants belonging to the Order Lepidoptera.

Order	Family	Genus	Species/Morphospecies	Overall abundance
Lepidoptera	Hesperiidae	Hylephila	1	2
	Lycaenidae	Celastrina	1	1
	Notodontidae	Josia	1	1
	Nymphalidae	Actinote	1	1
	Nymphalidae	Dione	1	2
	Pterophoridae		0	1
	Sphingidae	Aellopos	1	4

Supplementary Table 9: List of pollen samples used for DNA metabarcoding sequencing of the ITS2 region collected for each of the four fruit crops studied in San Gerardo.

**Order**	**Family**	**Lowest assignment**	**Group**	***Malus domestica***	***Persea americana***	***Prunus domestica***	***Rubus***
Coleoptera	Melyridae	*Astylus indentatus*	Other	0	0	1	2
Diptera	Anthomyiidae	*Hydrophoria sp1*	Non-Syrphidae	0	1	0	0
	Calliphoridae	NA	Non-Syrphidae	0	2	0	0
	Muscidae	*Limnophora sp1*	Non-Syrphidae	0	1	0	0
	Muscidae	*Limnophora sp3*	Non-Syrphidae	0	0	0	1
	Sarcophagidae	NA	Non-Syrphidae	0	2	0	0
	Sciaridae	NA	Non-Syrphidae	0	2	0	0
	Syrphidae	*Allograpta neotropica*	Syrphidae	0	6	0	0
	Syrphidae	*Allograpta sp8*	Syrphidae	0	1	0	0
	Syrphidae	*Argentinomyia sp1*	Syrphidae	0	1	0	0
	Syrphidae	*Argentinomyia sp2*	Syrphidae	0	1	0	0
	Syrphidae	NA	Syrphidae	0	11	0	0
	Syrphidae	*Ocyptamus sp4*	Syrphidae	0	1	0	0
	Syrphidae	*Ocyptamus sp5*	Syrphidae	0	1	0	0
	Tachinidae	*Epalpus sp1*	Non-Syrphidae	0	1	0	0
	Tachinidae	*Gaediopsis sp2*	Non-Syrphidae	0	1	0	0
	Tachinidae	*Jurinella sp1*	Non-Syrphidae	0	1	0	0
Hymenoptera	Apidae	*Apis mellifera*	Apis	26	7	20	17
	Apidae	*Bombus ephippiatus*	Bombus	10	6	0	26
	Apidae	*Partamona grandipennis*	Other	0	0	0	3
	Halictidae	*Lasioglossum sp1*	Other	3	0	1	0
	Halictidae	*Lasioglossum sp4*	Other	0	0	1	0
			Totalcount	39	46	23	49

References

Cheng, T., Xu, C., Lei, L., Li, C., Zhang, Y., & Zhou, S. (2016). Barcoding the kingdom plantae: New PCR primers for ITS regions of plants with improved universality and specificity. *Molecular Ecology Resources*, *16*(1), 138–149. https://doi.org/10.1111/1755-0998.12438

Doyle, J. J., & Doyle, J. L. (1987). A rapid dna isolation procedure for small quantities of fresh leaf tissue. *Phytochemical bulletin.*

Jeunen, G.-J., Dowle, E., Edgecombe, J., von Ammon, U., Gemmell, N. J., & Cross, H. (2023). Crabs—a software program to generate curated reference databases for metabarcoding sequencing data. *Molecular Ecology Resources*, *23*(3), 725–738. https://doi.org/10.1111/1755-0998.13741

Robeson II, M. S., O'Rourke, D. R., Kaehler, B. D., Ziemski, M., Dillon, M. R., Foster, J. T., & Bokulich, N. A. (2021). RESCRIPt: Reproducible sequence taxonomy reference database management [Publisher: Public Library of Science]. *PLOS Computational Biology*, *17*(11), e1009581. https://doi.org/10.1371/journal.pcbi.1009581
